# Dual mTORC1/2 inhibition compromises cell defenses against exogenous stress potentiating Obatoclax-induced cytotoxicity in atypical teratoid/rhabdoid tumors

**DOI:** 10.1038/s41419-022-04868-9

**Published:** 2022-04-28

**Authors:** Ashlyn Parkhurst, Sabrina Z. Wang, Tyler R. Findlay, Kristen J. Malebranche, Arman Odabas, Jesse Alt, Micah J. Maxwell, Harpreet Kaur, Cody J. Peer, William D. Figg, Katherine E. Warren, Barbara S. Slusher, Charles G. Eberhart, Eric H. Raabe, Jeffrey A. Rubens

**Affiliations:** 1 Division of Pediatric Oncology, Johns Hopkins University, School of Medicine, 1800 Orleans St, Baltimore, MD 21287 USA; 2 Sidney Kimmel Comprehensive Cancer Center, Johns Hopkins University, School of Medicine, 1800 Orleans St, Baltimore, MD 21287 USA; 3 Division of Cell Biology, Johns Hopkins University, School of Medicine, 1800 Orleans St, Baltimore, MD 21287 USA; 4 Clinical Pharmacology Program, National Cancer Institute at the National Institutes of Health, 37 Convent Dr, Bethesda, MD 20892 USA; 5 Johns Hopkins Drug Discovery, Johns Hopkins University, School of Medicine, 1800 Orleans St, Baltimore, MD 21287 USA; 6 Department of Neurology, Johns Hopkins University, School of Medicine, 1800 Orleans St, Baltimore, MD 21287 USA; 7 Pediatric Oncology Branch, National Cancer Institute at the National Institutes of Health, 37 Convent Dr, Bethesda, MD 20892 USA; 8 Dana Farber Cancer Institute (KEW), 450 Brookline Ave, Boston, MA 02215 USA; 9 Division of Neuropathology, Johns Hopkins University, School of Medicine, 1800 Orleans St, Baltimore, MD 21287 USA

**Keywords:** CNS cancer, Apoptosis

## Abstract

Atypical teratoid/rhabdoid tumors (AT/RT) are the most common malignant brain tumors of infancy and have a dismal 4-year event-free survival (EFS) of 37%. We have previously shown that mTOR activation contributes to AT/RT’s aggressive growth and poor survival. Targeting the mTOR pathway with the dual mTORC1/2 inhibitor TAK-228 slows tumor growth and extends survival in mice bearing orthotopic xenografts. However, responses are primarily cytostatic with limited durability. The aim of this study is to understand the impact of mTOR inhibitors on AT/RT signaling pathways and design a rational combination therapy to drive a more durable response to this promising therapy. We performed RNASeq, gene expression studies, and protein analyses to identify pathways disrupted by TAK-228. We find that TAK-228 decreases the expression of the transcription factor NRF2 and compromises AT/RT cellular defenses against oxidative stress and apoptosis. The BH3 mimetic, Obatoclax, is a potent inducer of oxidative stress and apoptosis in AT/RT. These complementary mechanisms of action drive extensive synergies between TAK-228 and Obatoclax slowing AT/RT cell growth and inducing apoptosis and cell death. Combination therapy activates the integrative stress response as determined by increased expression of phosphorylated EIF2α, ATF4, and CHOP, and disrupts the protective NOXA.MCL-1.BIM axis, forcing stressed cells to undergo apoptosis. Combination therapy is well tolerated in mice bearing orthotopic xenografts of AT/RT, slows tumor growth, and extends median overall survival. This novel combination therapy could be added to standard upfront therapies or used as a salvage therapy for relapsed disease to improve outcomes in AT/RT.

## Introduction

Atypical teratoid/rhabdoid tumors (AT/RT) are the most common malignant brain tumors of infancy [1]. Despite intensive multimodality therapies, four-year event-free survival remains 37% [2]. The recent advent of precise molecular therapies has helped improve survival in a wide range of cancers. Identifying novel targets in AT/RT and testing the efficacy of small-molecule inhibitors of these targets may help improve this dismal survival.

AT/RT’s relatively stable genome makes identifying targets for molecular therapies especially challenging [3, 4]. The majority of AT/RT are transformed and driven by a single recurring mutation in the SWI/SNF-related matrix-associated actin-dependent regulator of chromatin B1 (*SMARCB1*, also known as *INI1, SNF5*, and *BAF47*), while a minority of tumors harbor a mutation in *SMARCA4* (*BRG1*) [5, 6]. These genetic abnormalities disrupt the SWI/SNF chromatin-remodeling complex, which results in dysregulated gene expression. Despite the otherwise stable genome, high-throughput genetic and epigenetic studies have identified considerable molecular heterogeneity, dividing AT/RT into 3 distinct molecular subgroups of tumors [3, 4]. Many preclinical studies have focused on testing the efficacy of therapies targeting molecular abnormalities in these different subgroups of tumors [4, 7–9].

In our previous studies, we identified activation of the phosphatidylinositol-3 kinase (PI3K) pathway across all subgroups of AT/RT [10, 11]. About 30% of cancers harbor activating mutations or show evidence of activation of the PI3K pathway [12]. This activation contributes to cancer cell growth and therapy resistance and is associated with poor survival [13–15]. The mammalian or mechanistic target of rapamycin (mTOR) is a serine/threonine kinase and a critical component of the PI3K family. mTOR signaling regulates cell survival, cell growth, metabolism, protein synthesis, and autophagy [16].

TAK-228 (also known as Sapanisertib, MLN0128, INK128) is a highly selective ATP-competitive inhibitor that binds to the catalytic domain of mTOR to fully inhibit mTORC1 and mTORC2 complexes [17]. Phase-I clinical trials in adults demonstrated that TAK-228 is well tolerated with dose-limiting toxicities (DLT), including stomatitis, fatigue, and urticaria [18–20]. TAK-228 slows AT/RT cell growth and extends survival as a single agent in mice bearing orthotopic xenografts [10]. However, mice still succumb to further tumor progression due to cytostatic responses to mTOR inhibitors, which allows cancer cells to recover and continue their aggressive growth. In this study, we identify signaling pathways most significantly disrupted by TAK-228 and design a rational combination therapy that induces a cytotoxic response in AT/RT to enhance the durability of this promising therapy.

## Materials/subjects and methods

### Cell lines and cell culture

Cell lines were validated by short tandem-repeat (STR) testing (Johns Hopkins Genetics Resources Core) and confirmed *mycoplasma*-free with frequent PCR testing. Cells were grown in a humidified 37 °C chamber with 5% CO_2_. Passage numbers were limited between 1 and 20 for each cell line. BT-12 (RRID:CVCL_M155), BT37 (RRID:CVCL_JL57), and CHLA06-ATRT (ATCC Cat# CRL-3038, RRID:CVCL_AQ42) are previously described [10, 11, 21]. CHLA02-ATRT (ATCC Cat# CRL-3020, RRID:CVCL_B045), and CHLA05-ATRT (RRID:CVCL_AQ41) were obtained from Children’s Hospital of Los Angeles. CHLA-266 (RRID:CVCL_M149) and BT-12 were obtained from the Children’s Oncology Group cell repository. BT37 was obtained from St. Jude’s Research Hospital and derived from a human xenograft. CHLA02-ATRT, CHLA05-ATRT, CHLA06-ATRT, and CHLA-266 were cultured in EF media and BT-12 and BT37 in RPMI media as previously described [22–25]. While cell lines are not fully characterized, BT-12, CHLA-266, and CHLA06 likely represent the ATRT-MYC subgroup of AT/RT, CHLA02, and CHLA05 ATRT-SHH, and BT37 ATRT-TYR [7, 26].

TAK-228, Obatoclax, Navitoclax, and Venetoclax were obtained from Adooq Bioscience (Irvine, CA; Cat# A11461, A10665, A10022, A12500), AZD5991 and S63845 were obtained from MedChemExpress (Monmouth Junction, NJ; Cat# HY-101533, HY-100741). All medications were dissolved in DMSO for in vitro experiments. TAK-228 was dissolved in 5% methylcellulose and Obatoclax was dissolved in PBS, 30% PEG400, 0.5% Tween-80, and 5% propylene glycol for in vivo experiments.

### RNA sequencing

Each cell line was treated with TAK-228 20 nM (IC_50_) for 4 h and total RNA was extracted from cell pellets using Qiagen (Germantown, MD) RNeasy mini kit and column DNase treatment. RNAseq libraries were prepared using Illumina (San Diego, CA) TruSeq stranded mRNA sample preparation kit following the manufacturer’s recommended procedure with minor modifications. Briefly, mRNA was purified from 15 ng of total RNA using oligo dT RNA purification magnetic beads. The purified mRNA was then fragmented at 94 °C for 8 minutes, primed with random primers, and converted to double- strand cDNA. The resulting cDNA was end-repaired, dA-tailed, and ligated to TruSeq adapter (with unique dual-index barcodes). Libraries were then PCR amplified using 15 cycles. The final libraries were quality-checked, quantitated, and pooled in equal molar ratios for sequencing on NovaSeq SP 100cycle flowcells for 2X50-bp sequencing. The experiment was completed in 4 AT/RT cell models with 3 biological replicates in each group/condition. Differential gene expression was determined as genes with an FDR < 0.1 and >25% change in gene expression. Data uploaded to the Gene Expression Omnibus (GEO) functional genomics data repository and are publicly available at GSE198514.

#### Pathway analysis

Genes were identified that met the following criteria: (1) RNA expression identified in more than 75% of samples. (2) Significant changes in gene expression as determined by FDR < 0.1. (3) More than 25% decrease in RNA expression after TAK-228 treatment compared with DMSO control. Overrepresentation analysis completed using Panther functional database with WebGestalt to identify pathways most significantly disrupted by TAK-228 treatment [27].

### Lentiviral transduction

Supplies for shNRF2 knockdown and lentiviral activation experiments were purchased from Santa Cruz Biotechnology (Dallas, TX). Cells were grown in 6-well plates to 50% confluence. Polybrene (#sc-134220) was added to a final concentration of 5 µg/mL. shNRF2 lentiviral particles (#sc-370330-V), control shRNA lentiviral particles (#sc-108080), NRF2 activation particles (#sc-400017-LAC), or control activation particles (#sc-437282) were added to cell mixture and incubated for 24 h. Transduced cells were selected with puromycin dihydrochloride (sc-108071) 2.5 µg/mL over 72 h.

### Western blots

Cells were lysed in RIPA buffer and protein concentrations were quantified using Bradford Assay (Bio-Rad; Hercules, CA; #5000201) as previously described [7]. Membranes were incubated in primary antibody diluted 1:1000 in 5% BSA and secondary antibodies conjugated to horseradish peroxidase (1:3500). Antibodies: cleaved PARP (#9541), NRF2 (#12721), MCL-1 (#5453), BCL-xL (#2764), BCL-2 (#15071), pEI2Fα (#3398), EI2Fα (#5324), ATF4 (#11815), CHOP (#2895), NOXA (#14766), BIM (#2933), pAKT Ser473 (#9271), AKT (#4691), pS6 (#4858), and S6 (#2216) from Cell Signaling Technologies (Danvers, MA), β-Actin (#47778) from (Santa Cruz Biotechnology). Densitometry was performed using ImageJ v1.440 software as previously described [7]. See Supplemental Material for full western blots.

### Quantitative real-time PCR

Total RNA was isolated from cultured cells using RNeasy mini kit, Qiagen (Venlo, Netherlands) and cDNA was produced using Iscript cDNA synethesis kit, Bio-Rad (Hercules, CA; #1708890). qRT-PCR was performed using TaqMan master mix, ThermoFisher (Waltham, MA; #4444557). Primers: DDIT3 (HS00358796_g1), BOK (Hs011006404_m1), NOXA1 (Hs00611456_g1), HMOX1 (#Hs00157965_m1), NQO1 (#Hs01045993_g1), GCLM (#Hs00978072_m1), NFE2L2 (Hs00975961_g1), and POLR2A (Hs00172187_m1) were purchased from ThermoFisher. POLR2A was used as the endogenous control. The relative fold change was calculated based on the formula R = 2^−^^(Δ*Ct* sample − Δ*Ct* control)^.

### Glutathione-detection assay

Relative concentrations of intracellular reduced glutathione were determined as previously described [28]. Cells were plated in a 96-well plate. Hoechst 33342 stained DNA at a final concentration of 0.5 µg/mL by incubating with cells 30 minutes, 37 °C. Media was aspirated and 200 µL of 40 µM monochlorobimane added. Cells incubated for 30 minutes, 37 °C, and Hoechst stain intensity evaluated on plate reader (340em, 450) and Monochlorobimane (397em, 490). Relative concentrations of reduced glutathione were determined by the ratio of Monochlorobimane:Hoechst. Six to nine biological replicates included in each group as depicted in graphs.

### Metabolomics

Analyses on Agilent 1290 liquid chromatography system coupled to Agilent 6520 quadrupole time-of- flight mass spectrometer as previously described [29]. The mass spectrometer, equipped with a dual- electrospray ionization source, was run in negative ion and then positive ion mode. The scan range was 50–1600 m/z. Source settings; drying gas flow rate: 11 L/min; nebulizer: 40 pounds per square inch gauge; gas temperature: 350 °C; capillary voltage: 3000 V (neg), 2500 V (pos). Metabolites were identified using MS/MS, with fragments compared against Agilent Metlin Metabolomics Database and Library. Liquid chromatography–mass spectrometry data were analyzed using Agilent Qualitative Analysis B.07.00, El-MAVEN (Elucidata), and Metabolomic Analysis and Visualization ENgine (MAVEN). Five biological replicates completed in each treatment group.

### Cell death, growth, and oxidative stress assays

Assays were performed with MUSE ANNEXIN V & Dead Cell Kit (Luminex; Austin, Tx; MCH100105), MUSE Count & Viability Assay Kit (Luminex, MCH100102), and MUSE Oxidative Stress Kit (Luminex, MCH100111). Cells were plated in 6-well plates (200,000 cells/well). Adherent cell lines were incubated with trypsin at 37 °C with 5% CO_2_ for 5 minutes. Assay reagents were used per manufacturer’s guidelines and analyzed on MUSE cell analyzer (version 2). Three technical replicates in each group/model.

### Intracranial xenograft tumors

Animal experiments were conducted per NIH guidelines [30] for animal welfare and procedures were approved by the Johns Hopkins Animal Care and Use Committee, in compliance with United States Animal Welfare Act regulations and Public Health Service Policy. Intracranial xenografts were produced in anesthetized animals as previously described [10]. Four-to-six-week-old female Nu/Nu mice were obtained from Charles River Laboratories. About 1.0 × 10^5^ (BT37) or 2.5 × 10^4^ (CHLA06-ATRT) of viable cells were suspended in 5 µL of media and injected in the right frontal cortex.

### Pharmacokinetics

A validated ultra-high-performance liquid chromatography–tandem mass spectrometry (uHPLC–MS/MS) method was employed to assay concentrations of TAK-228 and Obatoclax in mouse whole blood and tissues. Tissue specimens were homogenized (100 mg/mL). TAK-228 or Obatoclax was extracted from blood or tissue homogenate with 14x (v/v) methyl t-butyl ether containing internal standard. The organic phase was evaporated, reconstituted, and injected into uHPLC–MS/MS. Analytes were chromatographically separated and isolated with Acquity^®^ UPLC system (Waters Corporation, Milford, MA) with a Polaris 3 C18-A, 2.0 × 50.0-mm analytical column (Agilent Technologies, Santa Clara, CA). An isocratic mobile phase of (65/35, v/v) 0.1% formic acid in water and 0.1% formic acid in acetonitrile was used at flow rate 0.2 mL/min. Analytes were detected (MS/MS) on Sciex QTRAP® 5500 System (Foster City, CA) with multiple-reaction monitoring (MRM); positive-mode ionization with mass transition (*m/z* 310.2 → 268.1); the IS was monitored using *m/z* 383.4 → 341.1. Calibration curve was modeled using linear regression, 1/x^2^ weighting, “x” is the ratio of analyte:internal standard concentration. The calibrated range for the blood assay was 1–1000 ng/mL (0.32–323 nM); tissue assay 5–12500 pg/mg (0.16–404 nM). The method meets FDA guidelines for linearity, accuracy, inter- and intrarun precision, 24-hour autosampler stability, and freeze–thaw stability [31]. Bailer’s method for destructive sampling was used to calculate area under the curve for each matrix analyzed [32]. The elimination rate (K_el_) was calculated based on natural log-transformed mean concentration values during elimination phase, half-life (T_1/2_) equaling ln(2)/K_el_. Clearance from blood was dose-divided by the AUC, and V_z_ was calculated as the clearance divided by K_el_. An unpaired two-tailed Z test (α = .05) was used to determine the statistical difference between AUCs in different tissue types [33]. In the TAK-228 pharmacokinetics experiment, three mice were included at each time point (hour 0, 2, 4, 8, and 24) and the experiment was completed with CHLA06 orthotopic tumors. CNS concentrations were determined from 4 different areas of the brain (brain stem, left cerebral hemisphere, right cerebral hemisphere, and cerebellum). The Obatoclax pharmacokinetics experiment measured concentrations of Obatoclax in the left and right cerebral cortex, cerebellum, and brain stem. Drug concentrations were measured at hours 0, 1, 2, 4, 8, and 24 after Obatoclax treatment.

### Pharmacodynamics

CHLA06 orthotopic xenografts were established in 16 4–6-week-old Nu/Nu female mice. Two mice were euthanized at time points between 0 and 72 h. Brains were dissected and tumors were extracted, and cells were lysed as described above. Western blot was performed for phospho-AKT ser473, total AKT, phospho-S6, and total S6. The experiment repeated after a single dose of vehicle, TAK-228 6 mg/kg oral, Obatoclax 6 mg/kg IP, and combination, tumors extracted, and western blot performed.

### Survival studies

Orthotopic tumors were established in 4–6-week-old Nu/Nu female mice as described above. Bioluminescence imaging was completed at 2-week intervals starting 3 days after tumor injections. Mice were randomized to treatment groups to establish comparative baseline bioluminescence between each group. Treatment was started on day 4 after tumor injection and medications were administered as described in “Results”. Ten mice were treated in each group. Before the experiment was started, it was determined that mice would be eliminated from analysis if they lost >20% weight in the first 2 weeks after tumor was established, given that this time course is more consistent with surgical complications or infection rather than tumor growth. Animals were otherwise euthanized upon distress, poor grooming, or loss of 20% body mass. Our cell models form invasive, lethal tumors in 100% of mice injected with tumor cells. We assume a power of 80% and an alpha of 0.05, a sample size of 10 mice in each arm will detect a 20% difference in tumor response between groups. Investigators were not blinded to the treatment groups.

### Statistical analysis

Statistical analysis completed with GraphPad Prism (GraphPad Software, San Diego, CA) or Excel (Microsoft; Redmond, WA). Single-group comparisons with two-tailed unpaired *t*-test and multiple-group comparisons with one-way ANOVA. All data followed a normal distribution. Survival studies completed using Log-Rank test. *P*-values of <0.05 were considered significant. Synergy calculations were conducted per guidelines described by Chou and Talalay method of synergy [34]. Data were normally distributed.

## Results

### TAK-228 disrupts the apoptotic signaling pathway and oxidative stress response in AT/RT

**Fig. 1:**
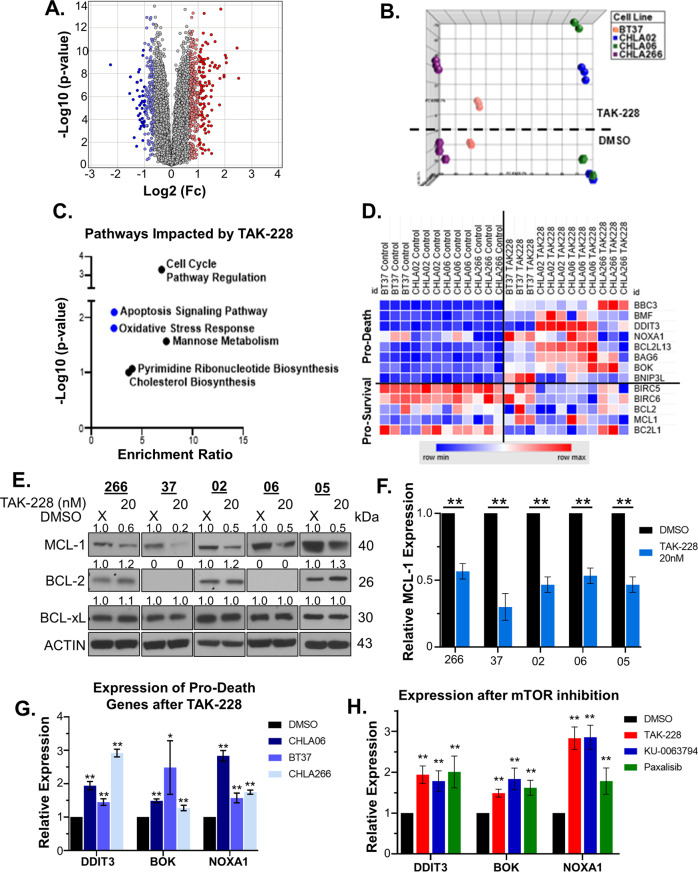
TAK-228 disrupts the apoptotic signaling pathway and oxidative stress response in AT/RT. **A** Volcano plot of the combined data over all cell models illustrates the impact of 4 h of TAK-228 20 nM treatment on gene expression. Genes included with FDR cutoff <0.1 and >25% change in expression. **B** Principal component analysis of gene expression profiles. Hexagons below the dashed line represent DMSO control-treated cells. Circles above the line represent TAK-228-treated cells. **C** Panther pathway analysis on genes downregulated after TAK-228 treatment identified signaling pathways most significantly disrupted by TAK-228 treatment. **D** Heatmap shows the most divergently expressed genes related to the apoptotic signaling pathway between DMSO and TAK-228-treated cells. Blue represents minimal expression and red maximum. RNASeq values normalized to the average value of the DMSO-treated samples in each cell model. **E** Western blot for anti-apoptotic proteins 24 h after TAK-228 20 nM treatment. Numbers above blots represent quantification of protein expression normalized to ACTIN. **F** Relative expression of MCL-1 as determined by quantification of western blots 24 h after TAK-228 treatment. MCL-1 expression is normalized to ACTIN and expressed as a ratio to DMSO control. **G** RT-PCR probing for pro-apoptotic genes *DDIT3, BOK*, and *NOXA1* 24 h after TAK-228. Significance measured in comparison with DMSO control. **H** Expression of pro-apoptotic genes 24 h after treatment with the mTOR/PI3K inhibitors TAK-228, KU-0063794, and Paxalisib dosed at the IC_50_ in CHLA06. Significance as compared with DMSO control. Results in this figure are presented as the mean +/− SEM, *p < 0.05 **p < 0.005, *t-*test.

We performed RNASeq 4 h after TAK-228 20 nM treatment to identify pathways most significantly disrupted by mTOR inhibition in AT/RT. This experiment was performed in cell models representing the 3 molecular subgroups of AT/RT (TYR: BT37, SHH: CHLA02, MYC: CHLA06, and CHLA-266) [7, 26]. TAK-228 affects the expression of numerous genes with both increases and decreases in expression (Fig. 1A, B, Supplementary Fig. 1A,B, Supplementary Table 1). These changes are most pronounced in the CHLA06 MYC model of AT/RT and more muted in the BT37 TYR model (Supplementary Fig. 1A), but each model has gene expression changes in response to TAK-228 treatment with many overlapping effects (Fig. 1B, Supplementary Fig. 1B). Overrepresentative pathway analysis of genes with significantly decreased expression after TAK-228 treatment identified, as expected, that cell cycle pathway regulation and metabolic pathways involved in cell growth like pyrimidine ribonucleotide biosynthesis and cholesterol biosynthesis are disrupted by mTOR inhibition (Fig. 1C, Supplementary Table 2). However, TAK-228 also disrupts the apoptotic signaling pathway and oxidative stress response, despite AT/RT having a mainly cytostatic response to therapy [35].

### mTOR inhibition primes cells for apoptosis

To understand how dual mTORC1/2 inhibition affects the apoptotic signaling pathway, we identified genes with the most significant changes in expression after TAK-228 treatment. In general, mTOR inhibition decreases prosurvival gene expression while increasing prodeath gene expression (Fig. 1D, Supplementary Table 3). We next evaluated changes in protein expression in key regulatory factors involved in the initiation of apoptosis. While TAK-228 has little impact on the expression of BCL-2 and BCL-xL, it decreases the expression of the anti-apoptotic protein MCL-1 (Fig. 1E, F). Changes in prosurvival gene expression were confirmed with RT-PCR (Fig. 1G). The dual mTORC1/2 inhibitor KU-0063794 and the PI3K inhibitor, Paxalisib (GDC-0084), which also inhibits both mTORC1 and mTORC2 activation, similarly increase expression of the pro-apoptotic genes, *DDIT3*, *BOK*, and *NOXA1* (Fig. 1H) [36, 37]. These findings suggest that inhibition of mTOR by antagonizing both mTORC1 and mTORC2 activation primes cells for apoptosis. However, mTOR inhibition alone does not induce high levels of apoptosis, suggesting that an additional prodeath signal is required to activate the signaling cascade.

### TAK-228 interferes with the AT/RT oxidative stress response

**Fig. 2:**
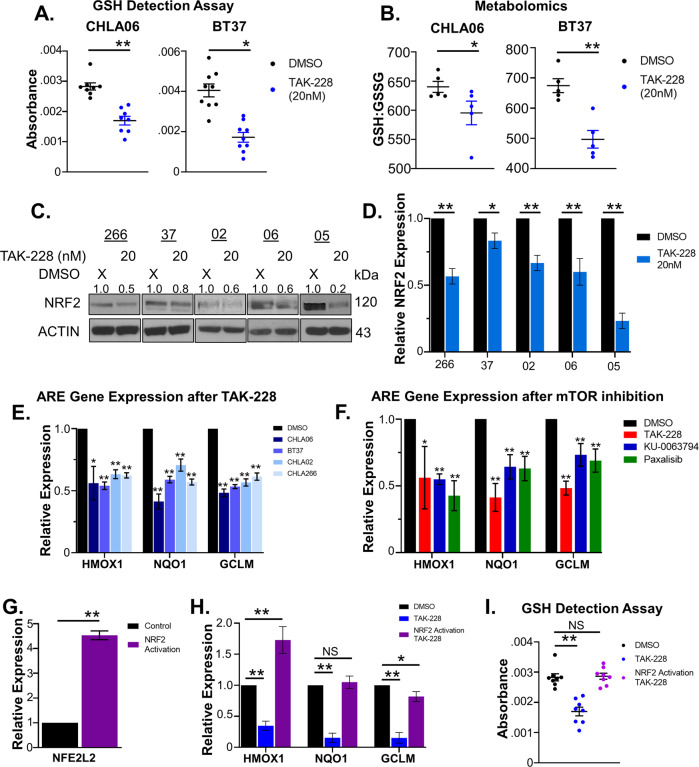
TAK-228 interferes with NRF2 transcriptional activity to disrupt the oxidative stress response in AT/RT. **A** Intracellular reduced glutathione as determined by the glutathione-detection assay. Higher absorbance represents higher intracellular glutathione concentrations. **B** Ratio of reduced glutathione to oxidized glutathione as determined by metabolomics analysis. **C** Western blot 24 h after TAK-228 probing for NRF2 expression. Numbers above blots represent quantification of protein expression normalized to ACTIN. **D** Relative expression of NRF2 as determined by quantification of western blots 24 h after TAK-228 treatment. NRF2 expression is normalized to ACTIN and expressed as a ratio to DMSO control. **E** Relative expression of NRF2-regulated ARE genes *HMOX1*, *NQO1*, and *GCLM* 24 h after TAK-228 compared with DMSO control. **F** NRF2-regulated ARE gene expression 24 h after treatment with TAK-228, KU-0063794, or Paxalisib compared with DMSO in CHLA06. **G** Expression of the NRF2 coding gene, *NFE2L2* after transduction of NRF2 lentiviral activation particles. **H** Expression of ARE genes after DMSO and TAK-228 treatment of empty vector control cells and after TAK-228 treatment of NRF2-activated cells. **I** Reduced intracellular glutathione after DMSO or TAK-228 treatment of empty vector control cells and after TAK-228 treatment of NRF2-activated cells. Results in this figure are presented as the mean +/− SEM, NS represents no significance, *p < 0.05, **p < 0.005, *t-*test.

Glutathione is the most abundant antioxidant in living organisms and is critical for cancer cell’s protection against reactive oxygen species (ROS) [38]. We measured the impact of TAK-228 on intracellular glutathione to confirm that TAK-228 disrupts the oxidative stress response. TAK-228 significantly decreases both intracellular concentrations of reduced glutathione and the ratio of reduced glutathione to oxidized glutathione, making cells more vulnerable to oxidative stress (Fig. 2A, B). NRF2 is a transcription factor, which regulates gene expression to coordinate cellular defenses against oxidative stress [39]. Preliminary data from early-phase clinical trials show that tumors with activating mutations in the NRF2 coding gene, *NFE2L2*, respond most significantly to TAK-228 [40]. Evaluation of a publicly available database of RNASeq on primary human AT/RT shows that *NFE2L2* is highly expressed in AT/RT with more than double the median expression compared with normal brain (Supplementary Fig. 2A) [41, 42]. Given that NRF2 is a possible biomarker predicting responses to TAK-228 and our findings that TAK-228 disrupts the oxidative stress response in AT/RT, we evaluated the direct effect of TAK-228 on NRF2. TAK-228 decreases expression of NRF2, most significant in CHL05, CHLA-266, and CHLA06 with more subtle effects in BT37 and CHLA02 (Fig. 2C, D). As a result, TAK-228 decreases the expression of NRF2-regulated antioxidant response elements (ARE) *HMOX1*, *NQO1*, and *GCLM* (Fig. 2E). Other mTOR/PI3K inhibitors, KU-0063794 and Paxalisib, have a similar impact on ARE gene expression (Fig. 2F). Short-hairpin knockdown of NRF2 decreases intracellular concentrations of glutathione (Supplementary Fig. 2B, C), while lentiviral activation of NRF2 (Fig. 2G) increases ARE gene expression and rescues the impact of TAK-228 on intracellular glutathione (Fig. 2H, I). These data demonstrate that TAK-228, through the inhibition of NRF2, disrupts AT/RT defenses against oxidative stress.

### Obatoclax induces oxidative stress and apoptosis in AT/RT

**Fig. 3:**
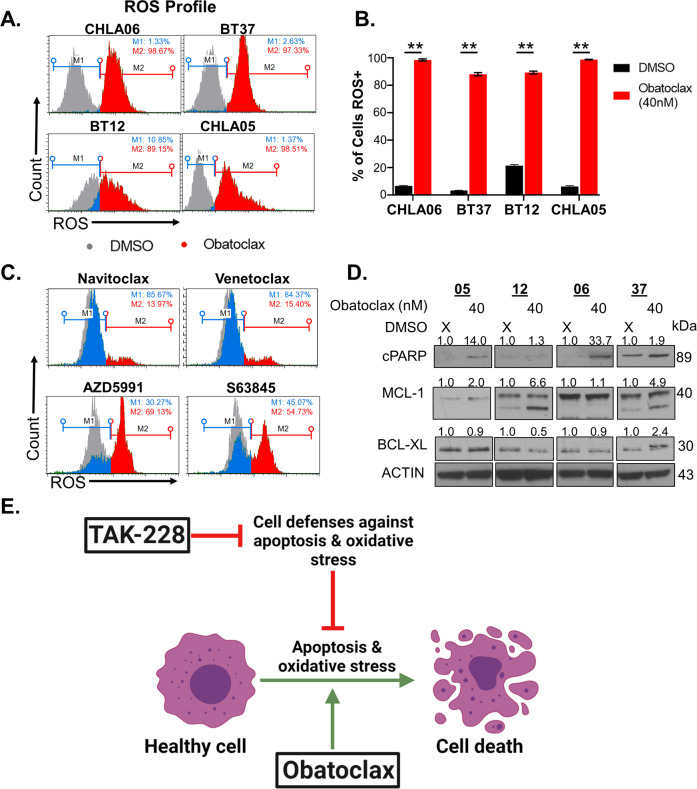
Obatoclax is a potent inducer of apoptosis and oxidative stress in AT/RT. **A** MUSE oxidative stress assay. M1 represents reactive oxygen species (ROS) negative cells, M2 ROS-positive cells. DMSO-treated cells shaded in gray, Obatoclax ROS negative (blue), ROS positive (red). **B** Graphs illustrating percent of cells ROS positive. The results are presented as the mean +/− SEM. **Indicates *p* < 0.005, *t-*test. **C** Muse oxidative stress assay. CHLA06 cells were treated with the IC_50_ dose of the BH3 mimetics Navitoclax and Venetoclax, and the MCL-1 inhibitors AZD5991 and S63845. ROS-negative cells (blue), ROS- positive cells (red). DMSO-treated cells shaded in gray. **D** Western blot 24 h after Obatoclax treatment in 4 AT/RT cell lines probing for cleaved PARP (cPARP) and the anti-apoptotic proteins MCL-1 and BCL-xL. Numbers above blots represent quantification of protein expression normalized to ACTIN. **E** Diagram illustrating complementary mechanisms of action between TAK-228 and Obatoclax. TAK-228 interferes with cell defenses against apoptosis and oxidative stress, while Obatoclax induces apoptosis and oxidative stress in AT/RT.

We next evaluated the potential for the BH3 mimetic, Obatoclax, to exploit these TAK-228-induced vulnerabilities in AT/RT. Obatoclax initiates apoptosis by binding and inhibiting the function of BCL-2 family anti-apoptotic proteins, freeing pro-apoptotic proteins to activate the apoptotic signaling cascade [43, 44]. Interestingly, we find that Obatoclax is a potent inducer of oxidative stress in AT/RT (Fig. 3A, B). Obatoclax is a pan-BCL-2 inhibitor of anti-apoptotic proteins, including MCL-1 [45]. Navitoclax and Venetoclax are BH3 mimetics that do not inhibit MCL-1, while AZD5991 and S63845 are MCL-1-specific inhibitors [46, 47]. Navitoclax and Venetoclax induce minimal oxidative stress in CHLA06, while MCL-1 inhibition with AZD5991 and S63845 induces oxidative stress similar to Obatoclax (Fig. 3C). Obatoclax also induces apoptosis in AT/RT cell models as determined by increased expression of cleaved PARP (Fig. 3D). This impact is especially notable in CHLA05 and CHLA06 but more muted in BT-12 and BT37. Obatoclax increases expression of MCL-1 in all cell models, but particularly in models more resistant to cell death. These data indicate that TAK-228 and Obatoclax have complementary mechanisms of action with TAK-228 compromising cell defenses against oxidative stress and apoptosis, while Obatoclax induces oxidative stress and apoptosis (Fig. 3E).

### TAK-228 combines with Obatoclax to reduce AT/RT cell viability and induce apoptosis

**Fig. 4:**
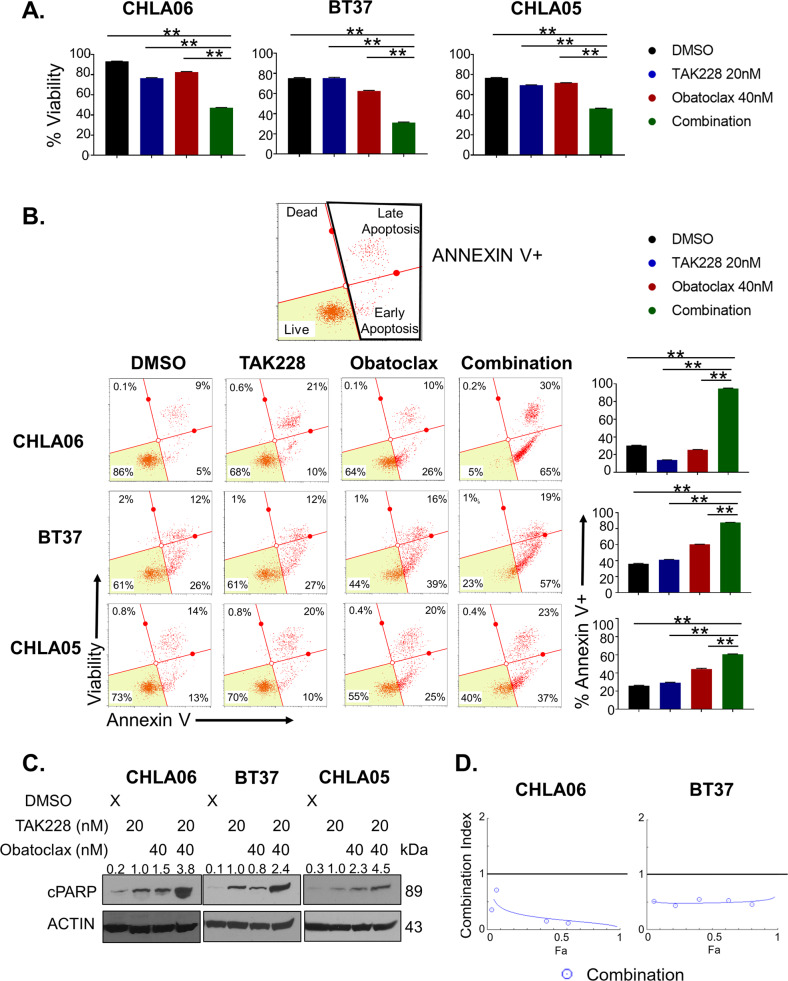
TAK-228 combines synergistically with Obatoclax to induce apoptosis and cell death in AT/RT. **A** Percent viability as determined by MUSE cell viability assay 72 h after TAK-228 20 nM combined with Obatoclax 40 nM compared with each medication alone and DMSO control. The results are presented as the mean +/− SEM, *p < 0.05 **p < 0.005, ANOVA. **B** Percent of cells undergoing apoptosis as determined by the ANNEXIN-V MUSE assay 24 h after treatment. Right side of the graph represents Annexin-V + cells, right lower-quadrant early apoptotic cells, upper-right quadrant late apoptotic cells. Graphs represent percent of cells Annexin-V positive. The results are presented as the mean +/− SEM, **p < 0.005, ANOVA. **C** Western blot for cleaved PARP expression 24 h after treatment. Numbers above blots represent quantification of protein expression normalized to ACTIN. **D** Formal synergy testing of fixed dose ratios of TAK-228 and Obatoclax by the CompuSyn Method of Synergy. Combination index <1 (below horizontal line on the graph) represents synergistic combinations.

To understand how these complementary mechanisms of action combine to impact AT/RT cell growth and survival, we treated AT/RT cell models with the combination of TAK-228/Obatoclax compared with each medication alone and DMSO controls. Combination therapy significantly decreases cell density and percent viability more than each medication alone (Fig. 3A, Supplementary Fig. 3A, B). Paxalisib and KU-0063794 have a similar effect combining with Obatoclax to decrease cell density and percent viability and increasing apoptosis (Supplementary Fig. 4A–D). TAK-228/Obatoclax combination therapy also significantly increases the number of cells undergoing apoptosis (Fig. 4B). This effect is greater than the additive effect of TAK-228 and Obatoclax alone and is most significant in the CHLA06 MYC model. Western blot for cleaved PARP confirmed that combination treatment activates apoptosis in AT/RT cell models (Fig. 4C). Formal synergy testing using the CompuSyn method of synergy indicates that this impact on apoptosis is highly synergistic in both the highly sensitive CHLA06 model and the more resistant BT37 tumor model [48] (Fig. 4D).

### The impact of TAK-228/Obatoclax is partially mediated through NRF2

**Fig. 5:**
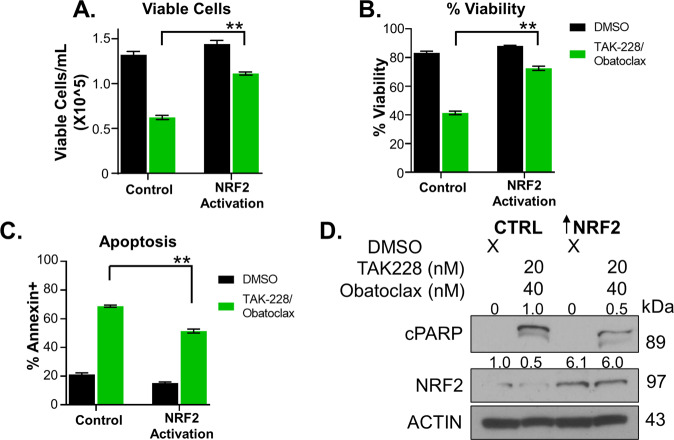
NRF2 rescues the impact of combination therapy on AT/RT growth, viability, and apoptosis. Empty vector control cells and NRF2-activated cells were treated with DMSO or TAK-228 combined with Obatoclax, and were compared with **A** number of viable cells in culture at 72 h, **B** percent viability at 72 h, **C** percent of cells ANNEXIN-V positive at 24 h, and **D** western blot probing for cleaved PARP and NRF2 expression 24 h after treatment. Numbers above blots represent quantification of protein expression normalized to ACTIN.

To understand how mTOR inhibition of NRF2 contributes to TAK-228/Obatoclax-mediated cell death, we rescued NRF2 transcriptional activity with lentiviral activation particles and compared the impact of TAK-228/Obatoclax treatment to empty vector-transduced cells. NRF2 activation protected cells against combination therapy with less reduction in both the number of viable cells in culture 72 h after treatment and in the percent viability of cells in culture (Fig. 5A, B). NRF2 activation also led to less induction of apoptosis 24 h after combination treatment compared with the same treatment of empty vector control cells (Fig. 5C, D). These studies demonstrate that decreases in NRF2 activity secondary to mTOR inhibition contribute to the impact of combination therapy on AT/RT cell growth, viability, and apoptosis.

### Combination therapy induces cell stress and compromises defenses against apoptosis

**Fig. 6:**
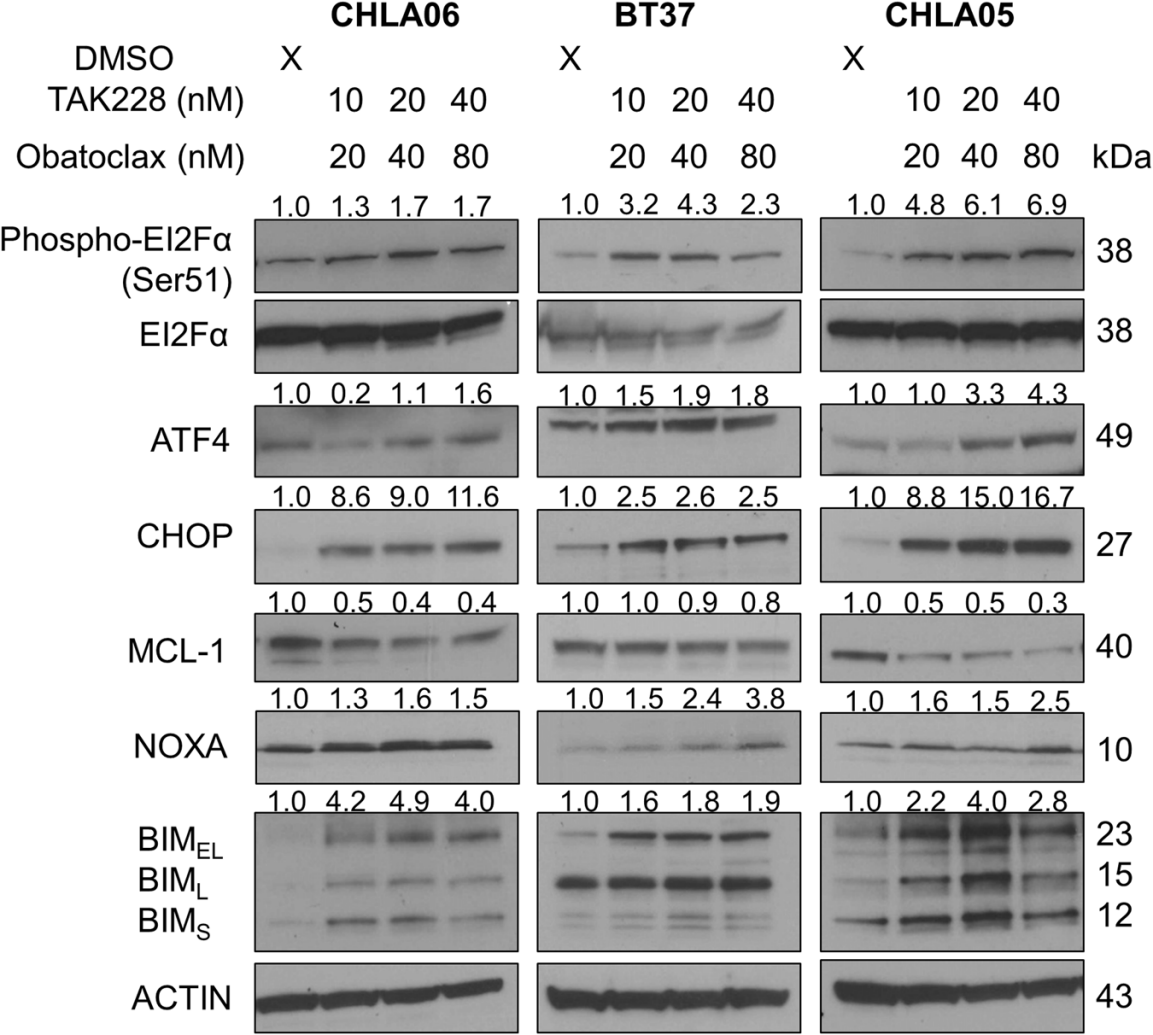
Combination therapy activates the integrative stress response and disrupts the NOXA.MCL-1.BIM axis, making stressed cells more prone to selection for apoptosis. Western blots in CHLA06, BT37, and CHLA05 cell models of AT/RT 24 h after DMSO control treatment and increasing fixed dose ratios of combination TAK-228 and Obatoclax treatment. Numbers above phospho-EI2Fα (Ser51) represent quantification of expression normalized to EI2Fα. Numbers above other blots represent quantification of protein expression normalized to ACTIN.

We next examined the mechanisms driving this cytotoxic response to combination therapy. Phosphorylation of EIF2α during periods of cellular stress leads to selective transcription of ATF4, which can increase the expression of the pro-apoptotic transcription factor, CHOP [49]. We find that increasing concentrations of combination therapy induce more cell stress as determined by expression of phosphorylated EIF2α and ATF4 (Fig. 6). As a result, CHOP expression also increases from baseline (Fig. 6). The NOXA–MCL-1–BIM axis serves as a gatekeeper determining which stressed cells undergo apoptosis [50–55]. Combination therapy disrupts this axis, increasing pro-apoptotic NOXA and BIM expression while decreasing anti-apoptotic MCL-1 expression. These studies demonstrate that TAK-228/Obatoclax induce cell death in AT/RT by both increasing cellular stress and disrupting defenses protecting cells from stress-induced cell death.

### TAK-228 and Obatoclax are rapidly absorbed and readily cross the blood–brain barrier

**Fig. 7:**
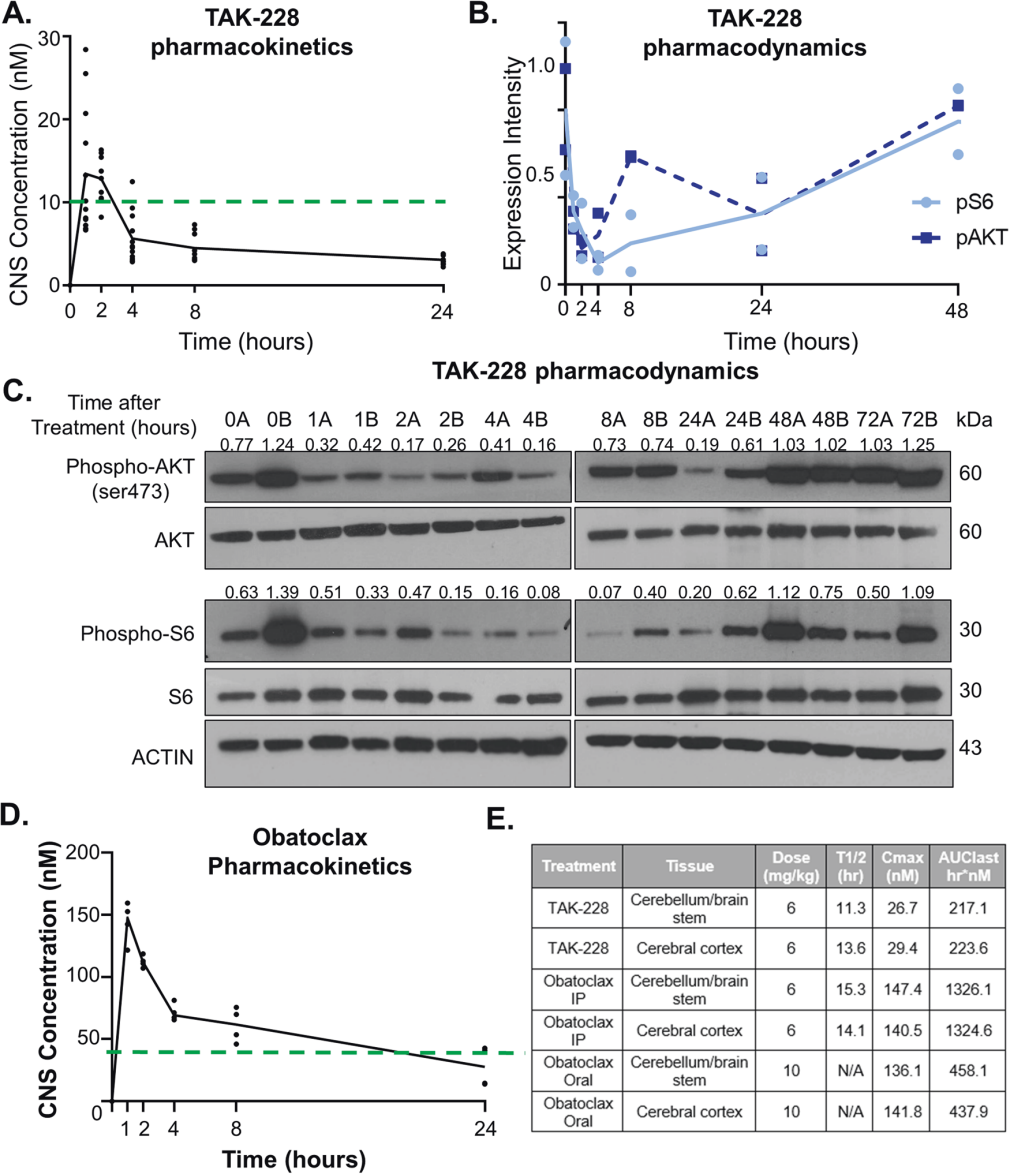
TAK-228 and Obatoclax readily cross the blood–brain barrier in supratherapeutic concentrations. Pharmacokinetic and pharmacodynamics studies in mice bearing AT/RT orthotopic xenografts after treatment with a single dose of 6 mg/kg oral TAK-228, 6 mg/kg IP Obatoclax, or 10 mg/kg oral Obatoclax. **A** TAK-228 concentrations in mouse brains 0–24 h after treatment. Circles represent each individual sample with line through the median values. Dotted horizontal line represents concentrations required in vitro to inhibit mTORC1 and mTORC2 activation. **B** Mice bearing orthotopic xenografts of CHLA06 were treated with a single dose of 6 mg/kg oral TAK-228 and euthanized between 0 and 72 h after treatment. Graphical representation of phospho-S6 and phospho-AKT Ser473 expression normalized to total S6 or AKT, respectively, and ACTIN and the mean value in untreated control tumors. Dotted line represents mean of phospho-AKT expression values and solid line represents mean of phospho-S6 expression levels. **C** Western blot of extracted tumor protein. Numbers above blot represent quantification of blots normalized to ACTIN and the mean of untreated control tumors. Two samples for each hour after treatment labeled A and B. **D** Obatoclax concentrations in mouse brains 0–24 h after 6 mg/kg IP treatment. Circle represents each individual sample with line connecting the median values. Dotted horizontal line represents concentrations required to reach IC_50_ in vitro. **E** Calculated half-life, maximum concentrations, and area under the curve in mouse brains.

The maximum tolerated dose (MTD) in clinical trials for TAK-228 is 30 mg when administered weekly [17, 18, 56]. Pharmacokinetic and pharmacodynamics analyses of the mouse equivalent to this weekly dose demonstrate that TAK-228 is rapidly absorbed and crosses the blood–brain barrier in concentrations exceeding the therapeutic threshold required to inhibit the mTOR pathway in vitro (Fig. 7A) [10]. Concentrations of TAK-228 in plasma and peripheral organs were 4x and 100x higher than in the CNS, respectively (Supplementary Fig. 5). TAK-228 inhibits activation of mTORC1/2 in the tumor field (CHLA06) as soon as 1 h after administration (Fig. 7B, C). Obatoclax, at equivalent dosing to the MTD identified in clinical trials [57, 58], is also rapidly absorbed and readily crosses the blood–brain barrier in concentrations exceeding concentrations required to inhibit 50% of cell growth in vitro (Fig. 7D). Oral administration of Obatoclax is absorbed more slowly but reaches similar concentrations to IP injections (Fig. 7E). TAK-228 and Obatoclax administered at the MTD equivalent therefore cross the blood–brain barrier to achieve adequate concentrations in the brain.

### Combination therapy improves survival in mice bearing orthotopic xenografts of AT/RT

**Fig. 8:**
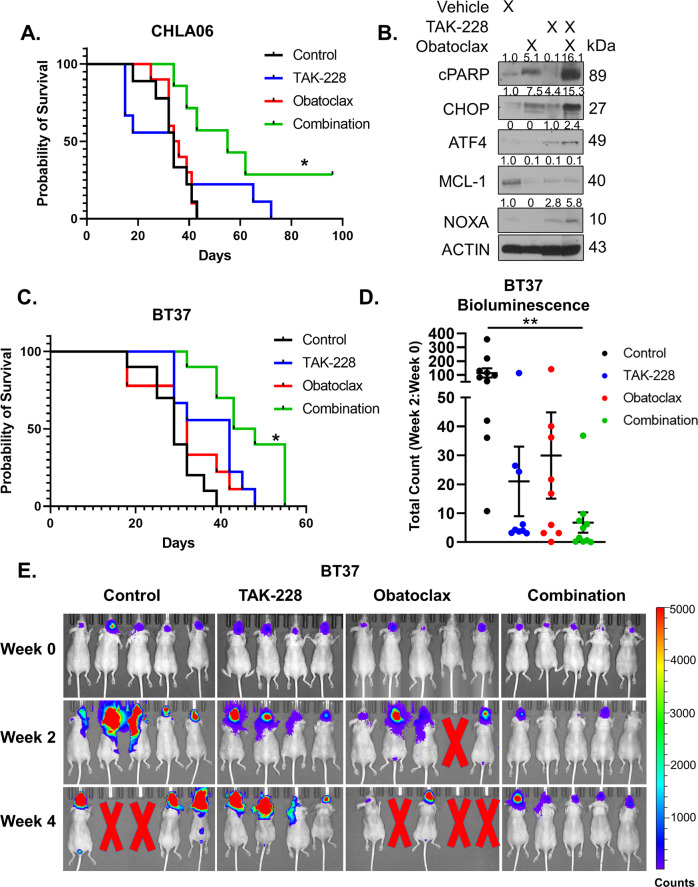
TAK-228 combines with Obatoclax to slow tumor growth and extend survival in mice bearing AT/RT orthotopic xenografts. **A** Kaplan–Meier curve: Survival of CHLA06 orthotopic AT/RT mouse models after treatment with TAK-228 combined with Obatoclax compared with each medication alone and vehicle controls. *p < 0.05, log-rank test for combination compared with control. **B** Western blot of tumors dissected from mouse brains 24 h after a single treatment of vehicle alone, Obatoclax 6 mg/kg IP, TAK-228 6 mg/kg oral, or Obatoclax 6 mg/kg IP combined with TAK-228 6 mg/kg oral. Numbers above blot represent quantification of blots normalized to ACTIN. **C** Kaplan–Meier curve showing survival of BT37 orthotopic AT/RT mouse models after treatment with TAK-228 combined with Obatoclax compared with each medication alone and vehicle controls. *p < 0.05, log-rank test compared with vehicle control. **D** Graph represents the ratio of BLI quantification after 2 weeks of treatment to baseline in each treatment group. The results are presented as the mean +/− SEM, **p < 0.005 by ANOVA. **E** Representative BLI in BT37 orthotopic mouse models at week 0, 2, and 4. Red X represents mice that died from tumor progression.

To determine the impact of combination therapy in a mammalian model of AT/RT, we developed orthotopic xenograft models with the CHLA06 and BT37 AT/RT cell models. In the CHLA06 experiment, mice were divided into 4 groups (Vehicle control, TAK-228 6 mg/kg oral once a week, Obatoclax 10 mg/kg oral once a week, and combination of TAK-228 and Obatoclax at the same doses administered weekly). Mice tolerated the treatment with no significant change in weight compared with vehicle controls (Supplementary Fig. 6A). Combination treatment significantly improved survival compared with vehicle control and each medication alone (Fig. 8A) with a median survival increasing from 34 to 55 days. About 25% of mice had a durable response to combination therapy. Bioluminescent imaging (BLI) demonstrated slower growth and a complete response in a subgroup of mice treated with combination treatment. However, some tumors continued to grow through combination treatment (Supplementary Fig. 6B). We next tested the effect of single treatments of vehicle, Obatoclax 6 mg/kg IP, TAK-228 10 mg/kg oral, or Obatoclax combined with TAK-228 at these same doses in CHLA06 orthotopic tumors. Obatoclax induced apoptosis as a single agent, while TAK-228 activated the integrative stress response and decreased MCL-1 expression (Fig. 8B). Combination therapy increased NOXA while reducing MCL-1 and induced high levels of apoptosis as determined by cleaved PARP expression (Fig. 8B). We next repeated the survival study in BT37 orthotopic xenograft models. Mice were divided in 4 groups in this experiment (Vehicle control, TAK-228 6 mg/kg oral once a week, Obatoclax 6 mg/kg intraperitoneal once a week, and combination of TAK-228 and Obatoclax at the same doses administered weekly). Given the more rapid absorption of Obatoclax when administered IP, we felt that IP dosing would combine more effectively with TAK-228 treatment administered at the same time point. This combination treatment significantly improved median survival from 29 to 44.5 days (Fig. 8C). Quantification of BLI after 2 weeks of treatment revealed slowing of tumor growth in combination-treated mice compared with vehicle control (Fig. 8D). BLI also demonstrated that some mice had significant regression in tumor size (Fig. 8E). Mice tolerated combination treatment without significant changes in weight (Supplementary Fig. 7). These studies demonstrate that combination therapy is well tolerated and extends survival in AT/RT.

## Discussion

The PI3K/mTOR pathway is a prime target for new cancer therapies. The pathway is frequently activated in aggressive cancers and regulates numerous signaling pathways contributing to cancer cell growth and survival [59, 60]. While initial responses to PI3K/mTOR pathway inhibition are encouraging, durability tends to be limited by cytostatic responses—slowing cancer cell growth but not inducing more permanent cell death [61]. In AT/RT, the dual mTORC1/2 inhibitor TAK-228 improves survival in mice bearing orthotopic xenografts, but mice eventually succumb to continued tumor growth [10]. Developing a better mechanistic understanding of cancer cell responses to mTOR inhibitors will help design improved treatment schemes to drive more durable responses to these promising therapies.

We find that the dual mTORC1/2 inhibitor, TAK-228, disrupts cell defenses against oxidative stress and apoptosis, making cancer cells more vulnerable to external stressors. This finding is in part due to decreases in NRF2 expression and activity. NRF2 is a cap’n’collar leukine zipper transcription factor that regulates the expression of genes involved in redox homeostasis, drug metabolism and excretion, energy metabolism, DNA repair, mitochondrial physiology, proliferation, and survival [62]. Clinical trials have shown that TAK-228 is most effective in treating cancers with constitutive activation of the NRF2 coding gene *NFE2L2* [40]. *NFE2L2* expression is higher in AT/RT compared with normal brain, which may help maintain a therapeutic index of response to mTOR inhibition [62–65]. NRF2 knockdown decreases the antioxidant glutathione, while activation of NRF2 restores ARE gene expression, increases intracellular glutathione after TAK-228 treatment, and partially rescues the effect of TAK-228/Obatoclax treatment on AT/RT cell growth and viability. While mTOR inhibitors alone do not induce cell death, they may sensitize cancer cells to cytotoxic therapies by disrupting NRF2 activity and cell defenses against oxidative stress and apoptosis.

We combine TAK-228 with the BH3 mimetic, Obatoclax, to induce a potent cytotoxic response in AT/RT. Obatoclax is a pan-BCL-2 family inhibitor of anti-apoptotic proteins, which was well-tolerated in clinical trials as a single-agent therapy [57, 58]. Toxicities are nonoverlapping with TAK-228 and include transient neurotoxicities such as infusion-related somnolence, ataxia, and confusion [57]. We find that Obatoclax and MCL-1-specific inhibitors induce high levels of oxidative stress in AT/RT. While MCL-1- specific antagonists tend to have poor CNS penetration [66], we find that Obatoclax readily crosses the blood–brain barrier in mouse models to achieve effective concentrations in the brain. Obatoclax combines with TAK-228 to induce substantial cell stress, activating the integrative stress response. The integrative stress response is primarily a protective pathway helping cells regain homeostasis in response to exogenous or endogenous stressors [53, 54]. However, if the stress is too intense or persists for too long, the integrative stress response can initiate apoptosis to eliminate the damaged cell [53, 54].

The NOXA.MCL-1.BIM axis helps regulate which cells undergo apoptosis in response to the integrative stress response [50–55]. NOXA is a pro-apoptotic protein, which selectively antagonizes MCL-1 by promoting its proteasomal degradation [50]. MCL-1 sequesters the pro-apoptotic BIM, so its degradation frees BIM to activate apoptosis and cell death [50, 67]. Therefore, the ratio of the pro-apoptotic factors (BIM and NOXA) to the anti-apoptotic factor MCL-1 helps understand which cells are most likely to undergo apoptosis in response to exogenous stress. Combination therapy decreases MCL-1 expression, while BIM and NOXA expression are increased. Therefore, this novel combination therapy induces high levels of apoptosis, both by stressing cancer cells and interfering with cell defenses protecting stressed cells from undergoing apoptosis. The resulting synergistic induction of apoptosis translates to orthotopic xenograft models with some complete responses to combination treatment and a significantly improved median survival compared with each medication alone and vehicle controls.

While there are some mixed results in these molecularly diverse cell models, the response to combination therapy tends to be more significant in the MYC subgroup of tumors. MYC drives rapid cell growth through the induction of anabolic and proliferative pathways [68]. As a by-product of this rapid growth, MYC induces considerable endogenous stress, which also activates the integrative stress response [68]. This endogenous stress may make the MYC subgroup more vulnerable to the additional exogenous stress induced by TAK-228/Obatoclax treatment.

The current study is limited by the few cell models that represent each molecular subgroup of tumors and the incomplete characterization of cell lines into molecular subgroups. It is important to more fully understand how the molecular diversity inherent to AT/RT impacts responses to therapy. In addition, orthotopic tumor responses to combination therapies were not durable with tumors continuing to grow and kill mice. Impact of radiation therapy on TAK-228/Obatoclax treatment would also be informative, given the use of radiation therapy in treatment of many patients with AT/RT. Further studies are required to identify mechanisms of therapeutic resistance and additional agents to help achieve a more durable response.

We have previously shown that TAK-228 also acts synergistically with platinum therapies to slow cell proliferation and induce cell death [10]. TAK-228/Obatoclax combination therapy could potentially be added into the current backbone of standard therapies [2] to drive a more durable response to therapy. While additional studies would be required to confirm its safety, the wide therapeutic index and minimally overlapping toxicities suggest that patients could safely tolerate the addition of TAK-228/Obatoclax into upfront therapies. Alternatively, TAK-228/Obatoclax therapy could be a therapeutic option to treat relapsed disease. Our findings demonstrating good CNS penetration for both the dual mTORC1/2 inhibitor, TAK-228 and the pan-BCL-2 family inhibitor, Obatoclax, and their efficacy in combination to extend survival in orthotopic models of AT/RT, demonstrate the potential of this novel combination therapy to improve outcomes in AT/RT.

## Supplementary information

Author Approval Emails

Reproducibility Checklist

Supplemental Figure 1

Supplemental Figure 2

Supplemental Figure 3

Supplemental Figure 4

Supplemental Figure 5

Supplemental Figure 6

Supplemental Table 1

Supplemental Table 2

Supplemental Table 3

Full WB

## Data Availability

The datasets used and analyzed in this study are available upon reasonable request.
