# Supplementary material for: Dual mTORC1/2 inhibition compromises cell defenses against exogenous stress potentiating Obatoclax-induced cytotoxicity in atypical teratoid/rhabdoid tumors
Source: Cell Death Dis. 2022 Apr 28;13(4):410. doi: 10.1038/s41419-022-04868-9 (PMC9050713; doi:10.1038/s41419-022-04868-9)
Supplement: Supplementary file 1 — Author Approval Emails [file 41419_2022_4868_MOESM1_ESM.pdf]

Jeffrey Rubens

From: Ashlyn Parkhurst <aparkhu1@gmail.com>
Sent: Friday, April 15, 2022 12:41 PM
To: Jeffrey Rubens
Subject: Re: Manuscript TAK-228/Obatoclax treatment in AT/RT - revisions due 3/16

Hi Jeff,

I approve Kristen to be added to the author list. So excited that it was accepted! Thanks for all the amazing work you did to get this project where it is today and for letting me be such a big part of it. I'm very proud of the work we were all able to accomplish together.

Thanks so much,

Ashlyn

On Mon, Apr 11, 2022 at 1:39 PM Jeffrey Rubens <jrubens6@jhmi.edu <mailto:jrubens6@jhmi.edu> > wrote:

Thank you all for your help with our manuscript "Dual mTORC1/2 inhibition compromises cell defenses against exogenous stress potentiating Obatoclax induced cytotoxicity in atypical teratoid/rhabdoid tumors". We had it provisionally accepted in Cell Death and Disease.

The journal is asking that everyone send back an email to me approving our change in authors. Kristen Malebranche was added to the author list after she contributed to the revisions of the manuscript. I also had mistakenly added her as division of pediatric oncology but changed her to division of cell biology in the final revision.

Please email me back your approval for Kristen to be added to the author list.

Thank you all again for your help.

Best wishes,

Jeff

Jeffrey Rubens, MD

Assistant Professor of Pediatrics & Oncology

Jeffrey Rubens

From: Sabrina Wang <sabrina.z.wang@gmail.com>
Sent: Monday, April 11, 2022 2:42 PM
To: Jeffrey Rubens
Subject: Re: Manuscript TAK-228/Obatoclax treatment in AT/RT - revisions due 3/16

Sounds great to me!
Sabrina

On Apr 11, 2022, at 2:39 PM, Jeffrey Rubens <jrubens6@jhmi.edu> wrote:

Thank you all for your help with our manuscript "Dual mTORC1/2 inhibition compromises cell defenses against exogenous stress potentiating Obatoclax induced cytotoxicity in atypical teratoid/rhabdoid tumors". We had it provisionally accepted in Cell Death and Disease.

The journal is asking that everyone send back an email to me approving our change in authors. Kristen Malebranche was added to the author list after she contributed to the revisions of the manuscript. I also had mistakenly added her as division of pediatric oncology but changed her to division of cell biology in the final revision.

Please email me back your approval for Kristen to be added to the author list.

Thank you all again for your help.

Best wishes,
Jeff

Jeffrey Rubens, MD
Assistant Professor of Pediatrics & Oncology
Division of Pediatric Oncology
Neuro-Oncology Service
Sidney Kimmel Cancer Center
Johns Hopkins University
Clinic: 410-955-8751
Fax: 410-955-0028
jrubens6@jhmi.edu

From: Jeffrey Rubens
Sent: Friday, March 11, 2022 10:11 AM
To: 'Ashlyn Parkhurst' <aparkhu1@gmail.com>; 'sabrina.z.wang@gmail.com' <sabrina.z.wang@gmail.com>; Tyler Findlay <tfindla1@jhu.edu>; 'Micah Maxwell' <micah.maxwell@gmail.com>; 'arman.odabas@nih.gov' <arman.odabas@nih.gov>; Jesse Alt <jalt1@jhmi.edu>; Harpreet Kaur <hkaur8@jhmi.edu>; 'cody.peer@nih.gov' <cody.peer@nih.gov>; 'figgw@mail.nih.gov' <figgw@mail.nih.gov>; 'KatherineE_Warren@DFCI.HARVARD.EDU' <KatherineE_Warren@DFCI.HARVARD.EDU>; Barbara Slusher <bslusher@jhmi.edu>; Charles Eberhart <ceberha@jhmi.edu>; Eric Raabe <eraabe2@jhmi.edu>; Kristen Malebranche <kmalebr1@jhmi.edu>
Subject: Manuscript TAK-228/Obatoclax treatment in AT/RT - revisions due 3/16

Jeffrey Rubens

From: Tyler Findlay
Sent: Monday, April 11, 2022 3:43 PM
To: Jeffrey Rubens
Subject: RE: Manuscript TAK-228/Obatoclax treatment in AT/RT - revisions due 3/16

Good afternoon Jeff,

I approve of the change in author, adding Kristen Malebranche as an author for the manuscript.

Best,

Tyler Findlay

From: Jeffrey Rubens <jrubens6@jhmi.edu>
Sent: Monday, April 11, 2022 2:39 PM
To: Ashlyn Parkhurst <aparkhu1@gmail.com>; sabrina.z.wang@gmail.com; Tyler Findlay <tfindla1@jhu.edu>; Micah Maxwell <micah.maxwell@gmail.com>; arman.odabas@nih.gov; Jesse Alt <jalt1@jhmi.edu>; Harpreet Kaur <hkaur8@jhmi.edu>; cody.peer@nih.gov; figgw@mail.nih.gov; KatherineE_Warren@DFCI.HARVARD.EDU; Barbara Slusher <bslusher@jhmi.edu>; Charles Eberhart <ceberha@jhmi.edu>; Eric Raabe <eraabe2@jhmi.edu>; Kristen Malebranche <kmalebr1@jhmi.edu>
Subject: RE: Manuscript TAK-228/Obatoclax treatment in AT/RT - revisions due 3/16

Thank you all for your help with our manuscript "Dual mTORC1/2 inhibition compromises cell defenses against exogenous stress potentiating Obatoclax induced cytotoxicity in atypical teratoid/rhabdoid tumors". We had it provisionally accepted in Cell Death and Disease.

The journal is asking that everyone send back an email to me approving our change in authors. Kristen Malebranche was added to the author list after she contributed to the revisions of the manuscript. I also had mistakenly added her as division of pediatric oncology but changed her to division of cell biology in the final revision.

Please email me back your approval for Kristen to be added to the author list.

Thank you all again for your help.

Best wishes,
Jeff

Jeffrey Rubens, MD
Assistant Professor of Pediatrics & Oncology
Division of Pediatric Oncology
Neuro-Oncology Service
Sidney Kimmel Cancer Center
Johns Hopkins University
Clinic: 410-955-8751
Fax: 410-955-0028
jrubens6@jhmi.edu

From: Jeffrey Rubens
Sent: Friday, March 11, 2022 10:11 AM

Jeffrey Rubens

**From:**	Micah Maxwell <micah.maxwell@gmail.com>
**Sent:**	Monday, April 11, 2022 2:48 PM
**To:**	Jeffrey Rubens
**Subject:**	Re: Manuscript TAK-228/Obatoclax treatment in AT/RT - revisions due 3/16

Approve

On Mon, Apr 11, 2022 at 2:39 PM Jeffrey Rubens <jrubens6@jhmi.edu> wrote:

Thank you all for your help with our manuscript "Dual mTORC1/2 inhibition compromises cell defenses against exogenous stress potentiating Obatoclax induced cytotoxicity in atypical teratoid/rhabdoid tumors". We had it provisionally accepted in Cell Death and Disease.

The journal is asking that everyone send back an email to me approving our change in authors. Kristen Malebranche was added to the author list after she contributed to the revisions of the manuscript. I also had mistakenly added her as division of pediatric oncology but changed her to division of cell biology in the final revision.

Please email me back your approval for Kristen to be added to the author list.

Thank you all again for your help.

Best wishes,

Jeff

Jeffrey Rubens, MD

Assistant Professor of Pediatrics & Oncology

Division of Pediatric Oncology

Neuro-Oncology Service

Sidney Kimmel Cancer Center

Johns Hopkins University

Jeffrey Rubens

From: Harpreet Kaur
Sent: Tuesday, April 12, 2022 3:09 AM
To: Jeffrey Rubens
Subject: Re: Manuscript TAK-228/Obatoclax treatment in AT/RT - revisions due 3/16

Hi Jeff,

Congratulations on the acceptance of the paper. I approve the addition of Kristen and any other change in authors.

Thanks,
Harpreet

Get Outlook for iOS

From: Jeffrey Rubens <jrubens6@jhmi.edu>
Sent: Tuesday, April 12, 2022 12:09:13 AM
To: Ashlyn Parkhurst <aparkhu1@gmail.com>; sabrina.z.wang@gmail.com <sabrina.z.wang@gmail.com>; Tyler Findlay <tfindla1@jhu.edu>; Micah Maxwell <micah.maxwell@gmail.com>; arman.odabas@nih.gov <arman.odabas@nih.gov>; Jesse Alt <jalt1@jhmi.edu>; Harpreet Kaur <hkaur8@jhmi.edu>; cody.peer@nih.gov <cody.peer@nih.gov>; figgw@mail.nih.gov <figgw@mail.nih.gov>; KatherineE_Warren@DFCI.HARVARD.EDU <KatherineE_Warren@DFCI.HARVARD.EDU>; Barbara Slusher <bslusher@jhmi.edu>; Charles Eberhart <ceberha@jhmi.edu>; Eric Raabe <eraabe2@jhmi.edu>; Kristen Malebranche <kmalebr1@jhmi.edu>
Subject: RE: Manuscript TAK-228/Obatoclax treatment in AT/RT - revisions due 3/16

Thank you all for your help with our manuscript "Dual mTORC1/2 inhibition compromises cell defenses against exogenous stress potentiating Obatoclax induced cytotoxicity in atypical teratoid/rhabdoid tumors". We had it provisionally accepted in Cell Death and Disease.

The journal is asking that everyone send back an email to me approving our change in authors. Kristen Malebranche was added to the author list after she contributed to the revisions of the manuscript. I also had mistakenly added her as division of pediatric oncology but changed her to division of cell biology in the final revision.

Please email me back your approval for Kristen to be added to the author list.

Thank you all again for your help.

Best wishes,
Jeff

Jeffrey Rubens, MD
Assistant Professor of Pediatrics & Oncology
Division of Pediatric Oncology
Neuro-Oncology Service
Sidney Kimmel Cancer Center
Johns Hopkins University
Clinic: 410-955-8751
Fax: 410-955-0028
jrubens6@jhmi.edu

Jeffrey Rubens

From: Jesse Alt
Sent: Tuesday, April 12, 2022 9:02 AM
To: Jeffrey Rubens
Subject: RE: Manuscript TAK-228/Obatoclax treatment in AT/RT - revisions due 3/16

Thanks Jeff,

I approve.

Jesse

From: Jeffrey Rubens <jrubens6@jhmi.edu>
Sent: Monday, April 11, 2022 2:39 PM
To: Ashlyn Parkhurst <aparkhu1@gmail.com>; sabrina.z.wang@gmail.com; Tyler Findlay <tfindla1@jhu.edu>; Micah Maxwell <micah.maxwell@gmail.com>; arman.odabas@nih.gov; Jesse Alt <jalt1@jhmi.edu>; Harpreet Kaur <hkaur8@jhmi.edu>; cody.peer@nih.gov; figgw@mail.nih.gov; KatherineE_Warren@DFCI.HARVARD.EDU; Barbara Slusher <bslusher@jhmi.edu>; Charles Eberhart <ceberha@jhmi.edu>; Eric Raabe <eraabe2@jhmi.edu>; Kristen Malebranche <kmalebr1@jhmi.edu>
Subject: RE: Manuscript TAK-228/Obatoclax treatment in AT/RT - revisions due 3/16

Thank you all for your help with our manuscript "Dual mTORC1/2 inhibition compromises cell defenses against exogenous stress potentiating Obatoclax induced cytotoxicity in atypical teratoid/rhabdoid tumors". We had it provisionally accepted in Cell Death and Disease.

The journal is asking that everyone send back an email to me approving our change in authors. Kristen Malebranche was added to the author list after she contributed to the revisions of the manuscript. I also had mistakenly added her as division of pediatric oncology but changed her to division of cell biology in the final revision.

Please email me back your approval for Kristen to be added to the author list.

Thank you all again for your help.

Best wishes,
Jeff

Jeffrey Rubens, MD
Assistant Professor of Pediatrics & Oncology
Division of Pediatric Oncology
Neuro-Oncology Service
Sidney Kimmel Cancer Center
Johns Hopkins University
Clinic: 410-955-8751
Fax: 410-955-0028
jrubens6@jhmi.edu

From: Jeffrey Rubens
Sent: Friday, March 11, 2022 10:11 AM
To: 'Ashlyn Parkhurst' <aparkhu1@gmail.com>; 'sabrina.z.wang@gmail.com' <sabrina.z.wang@gmail.com>; Tyler Findlay <tfindla1@jhu.edu>; 'Micah Maxwell' <micah.maxwell@gmail.com>; 'arman.odabas@nih.gov'

Jeffrey Rubens

From: armanodabas@gmail.com
Sent: Thursday, April 14, 2022 1:52 PM
To: Peer, Cody (NIH/NCI) [E]
Cc: Jeffrey Rubens
Subject: Re: Contact info for Arman Odabas

Hi Dr. Rubens,

Please feel free to add Kris Malebranche to the manuscript. Is there anything else I can help with?

Best,
Arman

On Apr 14, 2022, at 8:01 AM, Peer, Cody (NIH/NCI) [E] <cody.peer@nih.gov> wrote:

Hi Jeff,
I've copied Arman's personal email here. Hello Arman, I hope all is well.

Best,

Cody J. Peer, MS, PhD
Staff Scientist, Deputy Head
Clinical Pharmacology Program
National Cancer Institute
10 Center Dr, Room 5A03
Bethesda, MD 20892
Ph: 240-858-3204
M: 301-590-5497
https://ccr.cancer.gov/clinical-pharmacology-program

From: Jeffrey Rubens <jrubens6@jhmi.edu>
Sent: Thursday, April 14, 2022 7:43 AM
To: Peer, Cody (NIH/NCI) [E] <cody.peer@nih.gov>
Subject: [EXTERNAL] Contact info for Arman Odabas

CAUTION: This email originated from outside of the organization. Do not click links or open attachments unless you recognize the sender and are confident the content is safe.

Hi Cody,

Do you happen to have updated contact information for Arman Odabas? I need to get his approval for adding Kris Malebranche onto our manuscript. Thank you!

Jeff

Jeffrey Rubens

From: Peer, Cody (NIH/NCI) [E] <cody.peer@nih.gov>
Sent: Monday, April 11, 2022 2:46 PM
To: Jeffrey Rubens
Subject: Re: Manuscript TAK-228/Obatoclax treatment in AT/RT - revisions due 3/16

I approve.

From: "Jeffrey Rubens" <jrubens6@jhmi.edu>
Date: Monday, April 11, 2022 at 2:39:24 PM
To: "Ashlyn Parkhurst" <aparkhu1@gmail.com>, "sabrina.z.wang@gmail.com" <sabrina.z.wang@gmail.com>, "Tyler Findlay" <tfindla1@jhu.edu>, "Micah Maxwell" <micah.maxwell@gmail.com>, "arman.odabas@nih.gov" <arman.odabas@nih.gov>, "Jesse Alt" <jalt1@jhmi.edu>, "Harpreet Kaur" <hkaur8@jhmi.edu>, "Peer, Cody (NIH/NCI) [E]" <cody.peer@nih.gov>, "Figg, William (NIH/NCI) [E]" <figgw@mail.nih.gov>, "KatherineE_Warren@DFCI.HARVARD.EDU" <KatherineE_Warren@DFCI.HARVARD.EDU>, "bslusher" <bslusher@jhmi.edu>, "Charles Eberhart" <ceberha@jhmi.edu>, "Raabe, Eric" <eraabe2@jhmi.edu>, "Kristen Malebranche" <kmalebr1@jhmi.edu>
Subject: [EXTERNAL] RE: Manuscript TAK-228/Obatoclax treatment in AT/RT - revisions due 3/16

CAUTION: This email originated from outside of the organization. Do not click links or open attachments unless you recognize the sender and are confident the content is safe.

Thank you all for your help with our manuscript "Dual mTORC1/2 inhibition compromises cell defenses against exogenous stress potentiating Obatoclax induced cytotoxicity in atypical teratoid/rhabdoid tumors". We had it provisionally accepted in Cell Death and Disease.

The journal is asking that everyone send back an email to me approving our change in authors. Kristen Malebranche was added to the author list after she contributed to the revisions of the manuscript. I also had mistakenly added her as division of pediatric oncology but changed her to division of cell biology in the final revision.

Please email me back your approval for Kristen to be added to the author list.

Thank you all again for your help.

Best wishes,
Jeff

Jeffrey Rubens, MD
Assistant Professor of Pediatrics & Oncology
Division of Pediatric Oncology
Neuro-Oncology Service
Sidney Kimmel Cancer Center
Johns Hopkins University
Clinic: 410-955-8751
Fax: 410-955-0028
jrubens6@jhmi.edu

Jeffrey Rubens

From: Figg, William (NIH/NCI) [E] <figgw@mail.nih.gov>
Sent: Monday, April 11, 2022 3:05 PM
To: Jeffrey Rubens
Subject: Re: [EXTERNAL] RE: Manuscript TAK-228/Obatoclax treatment in AT/RT - revisions due 3/16

I fully support adding Kristen Malebranche as an author
Cheers!

William Douglas Figg, Sr
Senior Investigator/Head of the Clinical Pharmacology Program in the Office of the Clinical Director, CCR, NCI, NIH/Head of the Molecular Pharmacology Section, GMB, CCR, NCI, NIH/ Deputy Branch Chief of the Genitourinary Malignancies Branch, CCR, NCI, NIH/Co-Head of the Office of Translational Resources, CCR, NCI, NIH/Associate Director of the Center for Cancer Research, National Cancer Institute, National Institutes of Health 9000 Rockville Pike, Bldg 10/Room 5A03, Bethesda, MD 20892, O) +1 240 760 6179, M) +1 240 338 6898, figgw@helix.nih.gov, wdfigg@hotmail.com

---

From: Jeffrey Rubens <jrubens6@jhmi.edu>
Date: Monday, April 11, 2022 at 2:39 PM
To: Ashlyn Parkhurst <aparkhu1@gmail.com>, "sabrina.z.wang@gmail.com" <sabrina.z.wang@gmail.com>, Tyler Findlay <tfindla1@jhu.edu>, Micah Maxwell <micah.maxwell@gmail.com>, Arman Odabas <arman.odabas@nih.gov>, Jesse Alt <jalt1@jhmi.edu>, Harpreet Kaur <hkaur8@jhmi.edu>, Cody Peer <cody.peer@nih.gov>, "Figg, William (NIH/NCI) [E]" <figgw@mail.nih.gov>, "KatherineE_Warren@DFCI.HARVARD.EDU" <KatherineE_Warren@DFCI.HARVARD.EDU>, bslusher <bslusher@jhmi.edu>, Charles Eberhart <ceberha@jhmi.edu>, "Raabe, Eric" <eraabe2@jhmi.edu>, Kristen Malebranche <kmalebr1@jhmi.edu>
Subject: [EXTERNAL] RE: Manuscript TAK-228/Obatoclax treatment in AT/RT - revisions due 3/16

CAUTION: This email originated from outside of the organization. Do not click links or open attachments unless you recognize the sender and are confident the content is safe.

Thank you all for your help with our manuscript "Dual mTORC1/2 inhibition compromises cell defenses against exogenous stress potentiating Obatoclax induced cytotoxicity in atypical teratoid/rhabdoid tumors". We had it provisionally accepted in Cell Death and Disease.

The journal is asking that everyone send back an email to me approving our change in authors. Kristen Malebranche was added to the author list after she contributed to the revisions of the manuscript. I also had mistakenly added her as division of pediatric oncology but changed her to division of cell biology in the final revision.

Please email me back your approval for Kristen to be added to the author list.

Thank you all again for your help.

Best wishes,
Jeff

Jeffrey Rubens, MD
Assistant Professor of Pediatrics & Oncology
Division of Pediatric Oncology
Neuro-Oncology Service

Jeffrey Rubens

**From:**	Warren, Katherine E. <KatherineE_Warren@DFCI.HARVARD.EDU>
**Sent:**	Thursday, April 14, 2022 9:47 AM
**To:**	Jeffrey Rubens
**Subject:**	Re: Manuscript TAK-228/Obatoclax treatment in AT/RT - revisions due 3/16

Sorry for the delay, Jeff.

Yes, I approve of adding Kris Malebranche as a co-author.

Kathy

From: Jeffrey Rubens <jrubens6@jhmi.edu>
Date: Thursday, April 14, 2022 at 7:41 AM
To: "KatherineE_Warren@dfci.harvard.edu" <KatherineE_Warren@DFCI.HARVARD.EDU>
Subject: FW: Manuscript TAK-228/Obatoclax treatment in AT/RT - revisions due 3/16

External Email - Use Caution

Hi Kathy, I hope you have been well. Could you send me an email expressing your approval for adding Kris Malebranche onto the author list for the TAK-228/Obatoclax manuscript? Thank you!

Jeff

From: Jeffrey Rubens
Sent: Monday, April 11, 2022 2:39 PM
To: Ashlyn Parkhurst <aparkhu1@gmail.com>; sabrina.z.wang@gmail.com; Tyler Findlay <tfindla1@jhu.edu>; Micah Maxwell <micah.maxwell@gmail.com>; arman.odabas@nih.gov; Jesse Alt <jalt1@jhmi.edu>; Harpreet Kaur <hkaur8@jhmi.edu>; cody.peer@nih.gov; figgw@mail.nih.gov; KatherineE_Warren@DFCI.HARVARD.EDU; Barbara Slusher <bslusher@jhmi.edu>; Charles Eberhart <ceberha@jhmi.edu>; Eric Raabe <eraabe2@jhmi.edu>; Kristen Malebranche <kmalebr1@jhmi.edu>
Subject: RE: Manuscript TAK-228/Obatoclax treatment in AT/RT - revisions due 3/16

Thank you all for your help with our manuscript "Dual mTORC1/2 inhibition compromises cell defenses against exogenous stress potentiating Obatoclax induced cytotoxicity in atypical teratoid/rhabdoid tumors". We had it provisionally accepted in Cell Death and Disease.

The journal is asking that everyone send back an email to me approving our change in authors. Kristen Malebranche was added to the author list after she contributed to the revisions of the manuscript. I also had mistakenly added her as division of pediatric oncology but changed her to division of cell biology in the final revision.

Please email me back your approval for Kristen to be added to the author list.

Thank you all again for your help.

Best wishes,

Jeffrey Rubens

From: Barbara Slusher
Sent: Monday, April 11, 2022 3:15 PM
To: Jeffrey Rubens
Subject: RE: Manuscript TAK-228/Obatoclax treatment in AT/RT - revisions due 3/16

I approve Kristen Malebranche to be added to the author list

From: Jeffrey Rubens
Sent: Monday, April 11, 2022 2:39 PM
To: Ashlyn Parkhurst <aparkhu1@gmail.com>; sabrina.z.wang@gmail.com; Tyler Findlay <tfindla1@jhu.edu>; Micah Maxwell <micah.maxwell@gmail.com>; arman.odabas@nih.gov; Jesse Alt <jalt1@jhmi.edu>; Harpreet Kaur <hkaur8@jhmi.edu>; cody.peer@nih.gov; figgw@mail.nih.gov; KatherineE_Warren@DFCI.HARVARD.EDU; Barbara Slusher <bslusher@jhmi.edu>; Charles Eberhart <ceberha@jhmi.edu>; Eric Raabe <eraabe2@jhmi.edu>; Kristen Malebranche <kmalebr1@jhmi.edu>
Subject: RE: Manuscript TAK-228/Obatoclax treatment in AT/RT - revisions due 3/16

Thank you all for your help with our manuscript "Dual mTORC1/2 inhibition compromises cell defenses against exogenous stress potentiating Obatoclax induced cytotoxicity in atypical teratoid/rhabdoid tumors". We had it provisionally accepted in Cell Death and Disease.

The journal is asking that everyone send back an email to me approving our change in authors. Kristen Malebranche was added to the author list after she contributed to the revisions of the manuscript. I also had mistakenly added her as division of pediatric oncology but changed her to division of cell biology in the final revision.

Please email me back your approval for Kristen to be added to the author list.

Thank you all again for your help.

Best wishes,
Jeff

Jeffrey Rubens, MD
Assistant Professor of Pediatrics & Oncology
Division of Pediatric Oncology
Neuro-Oncology Service
Sidney Kimmel Cancer Center
Johns Hopkins University
Clinic: 410-955-8751
Fax: 410-955-0028
jrubens6@jhmi.edu

From: Jeffrey Rubens
Sent: Friday, March 11, 2022 10:11 AM
To: 'Ashlyn Parkhurst' <aparkhu1@gmail.com>; 'sabrina.z.wang@gmail.com' <sabrina.z.wang@gmail.com>; Tyler Findlay <tfindla1@jhu.edu>; 'Micah Maxwell' <micah.maxwell@gmail.com>; 'arman.odabas@nih.gov' <arman.odabas@nih.gov>; Jesse Alt <jalt1@jhmi.edu>; Harpreet Kaur <hkaur8@jhmi.edu>; 'cody.peer@nih.gov' <cody.peer@nih.gov>; 'figgw@mail.nih.gov' <figgw@mail.nih.gov>; 'KatherineE_Warren@DFCI.HARVARD.EDU' <KatherineE_Warren@DFCI.HARVARD.EDU>; Barbara Slusher <bslusher@jhmi.edu>; Charles Eberhart

Jeffrey Rubens

From: Charles Eberhart
Sent: Monday, April 11, 2022 2:53 PM
To: Jeffrey Rubens
Subject: RE: Manuscript TAK-228/Obatoclax treatment in AT/RT - revisions due 3/16

I approve the author change

Charles Eberhart

From: Jeffrey Rubens <jrubens6@jhmi.edu>
Sent: Monday, April 11, 2022 2:39 PM
To: Ashlyn Parkhurst <aparkhu1@gmail.com>; sabrina.z.wang@gmail.com; Tyler Findlay <tfindla1@jhu.edu>; Micah Maxwell <micah.maxwell@gmail.com>; arman.odabas@nih.gov; Jesse Alt <jalt1@jhmi.edu>; Harpreet Kaur <hkaur8@jhmi.edu>; cody.peer@nih.gov; figgw@mail.nih.gov; KatherineE_Warren@DFCI.HARVARD.EDU; Barbara Slusher <bslusher@jhmi.edu>; Charles Eberhart <ceberha@jhmi.edu>; Eric Raabe <eraabe2@jhmi.edu>; Kristen Malebranche <kmalebr1@jhmi.edu>
Subject: RE: Manuscript TAK-228/Obatoclax treatment in AT/RT - revisions due 3/16

Thank you all for your help with our manuscript "Dual mTORC1/2 inhibition compromises cell defenses against exogenous stress potentiating Obatoclax induced cytotoxicity in atypical teratoid/rhabdoid tumors". We had it provisionally accepted in Cell Death and Disease.

The journal is asking that everyone send back an email to me approving our change in authors. Kristen Malebranche was added to the author list after she contributed to the revisions of the manuscript. I also had mistakenly added her as division of pediatric oncology but changed her to division of cell biology in the final revision.

Please email me back your approval for Kristen to be added to the author list.

Thank you all again for your help.

Best wishes,
Jeff

Jeffrey Rubens, MD
Assistant Professor of Pediatrics & Oncology
Division of Pediatric Oncology
Neuro-Oncology Service
Sidney Kimmel Cancer Center
Johns Hopkins University
Clinic: 410-955-8751
Fax: 410-955-0028
jrubens6@jhmi.edu

From: Jeffrey Rubens
Sent: Friday, March 11, 2022 10:11 AM
To: 'Ashlyn Parkhurst' <aparkhu1@gmail.com>; 'sabrina.z.wang@gmail.com' <sabrina.z.wang@gmail.com>; Tyler Findlay <tfindla1@jhu.edu>; 'Micah Maxwell' <micah.maxwell@gmail.com>; 'arman.odabas@nih.gov' <arman.odabas@nih.gov>; Jesse Alt <jalt1@jhmi.edu>; Harpreet Kaur <hkaur8@jhmi.edu>; 'cody.peer@nih.gov' <cody.peer@nih.gov>; 'figgw@mail.nih.gov' <figgw@mail.nih.gov>; 'KatherineE_Warren@DFCI.HARVARD.EDU'

Jeffrey Rubens

From: Eric Raabe
Sent: Monday, April 11, 2022 4:31 PM
To: Jeffrey Rubens
Subject: Re: Manuscript TAK-228/Obatoclax treatment in AT/RT - revisions due 3/16

I approve the addition of Kris as a coauthor.

Eric

Eric Raabe MD, PhD
Associate Professor of Pediatric Oncology
Johns Hopkins University School of Medicine
Sent from my iPhone

On Apr 11, 2022, at 2:39 PM, Jeffrey Rubens <jrubens6@jhmi.edu> wrote:

Thank you all for your help with our manuscript "Dual mTORC1/2 inhibition compromises cell defenses against exogenous stress potentiating Obatoclax induced cytotoxicity in atypical teratoid/rhabdoid tumors". We had it provisionally accepted in Cell Death and Disease.

The journal is asking that everyone send back an email to me approving our change in authors. Kristen Malebranche was added to the author list after she contributed to the revisions of the manuscript. I also had mistakenly added her as division of pediatric oncology but changed her to division of cell biology in the final revision.

Please email me back your approval for Kristen to be added to the author list.

Thank you all again for your help.

Best wishes,
Jeff

Jeffrey Rubens, MD
Assistant Professor of Pediatrics & Oncology
Division of Pediatric Oncology
Neuro-Oncology Service
Sidney Kimmel Cancer Center
Johns Hopkins University
Clinic: 410-955-8751
Fax: 410-955-0028
jrubens6@jhmi.edu

From: Jeffrey Rubens
Sent: Friday, March 11, 2022 10:11 AM
To: 'Ashlyn Parkhurst' <aparkhu1@gmail.com>; 'sabrina.z.wang@gmail.com' <sabrina.z.wang@gmail.com>; Tyler Findlay <tfindla1@jhu.edu>; 'Micah Maxwell' <micah.maxwell@gmail.com>; 'arman.odabas@nih.gov' <arman.odabas@nih.gov>; Jesse Alt
