# Supplementary figures and images for: Dual mTORC1/2 inhibition compromises cell defenses against exogenous stress potentiating Obatoclax-induced cytotoxicity in atypical teratoid/rhabdoid tumors
Source: Cell Death Dis. 2022 Apr 28;13(4):410. doi: 10.1038/s41419-022-04868-9 (PMC9050713; doi:10.1038/s41419-022-04868-9)

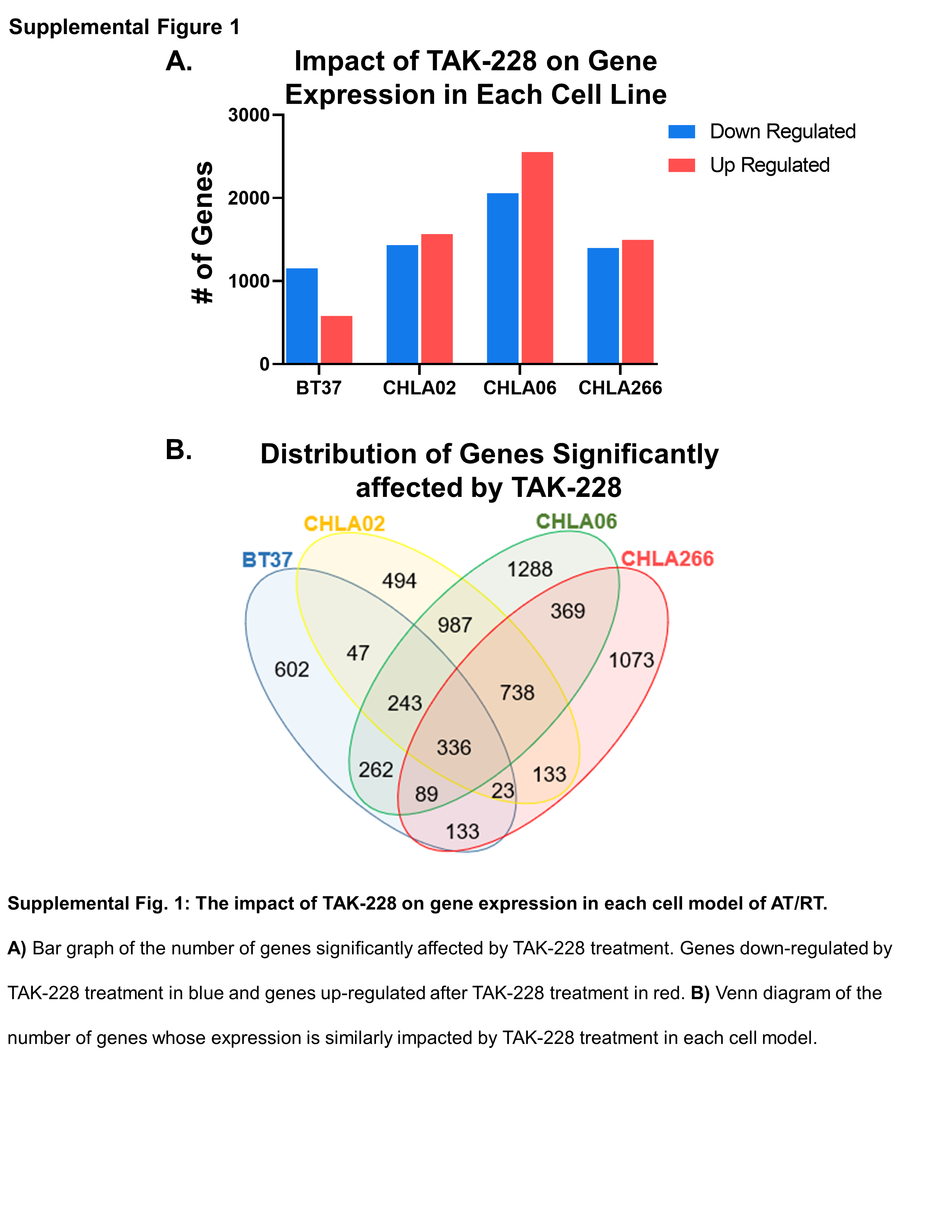

Supplement: Supplementary file 3 — Supplemental Figure 1 [file 41419_2022_4868_MOESM3_ESM.tif]

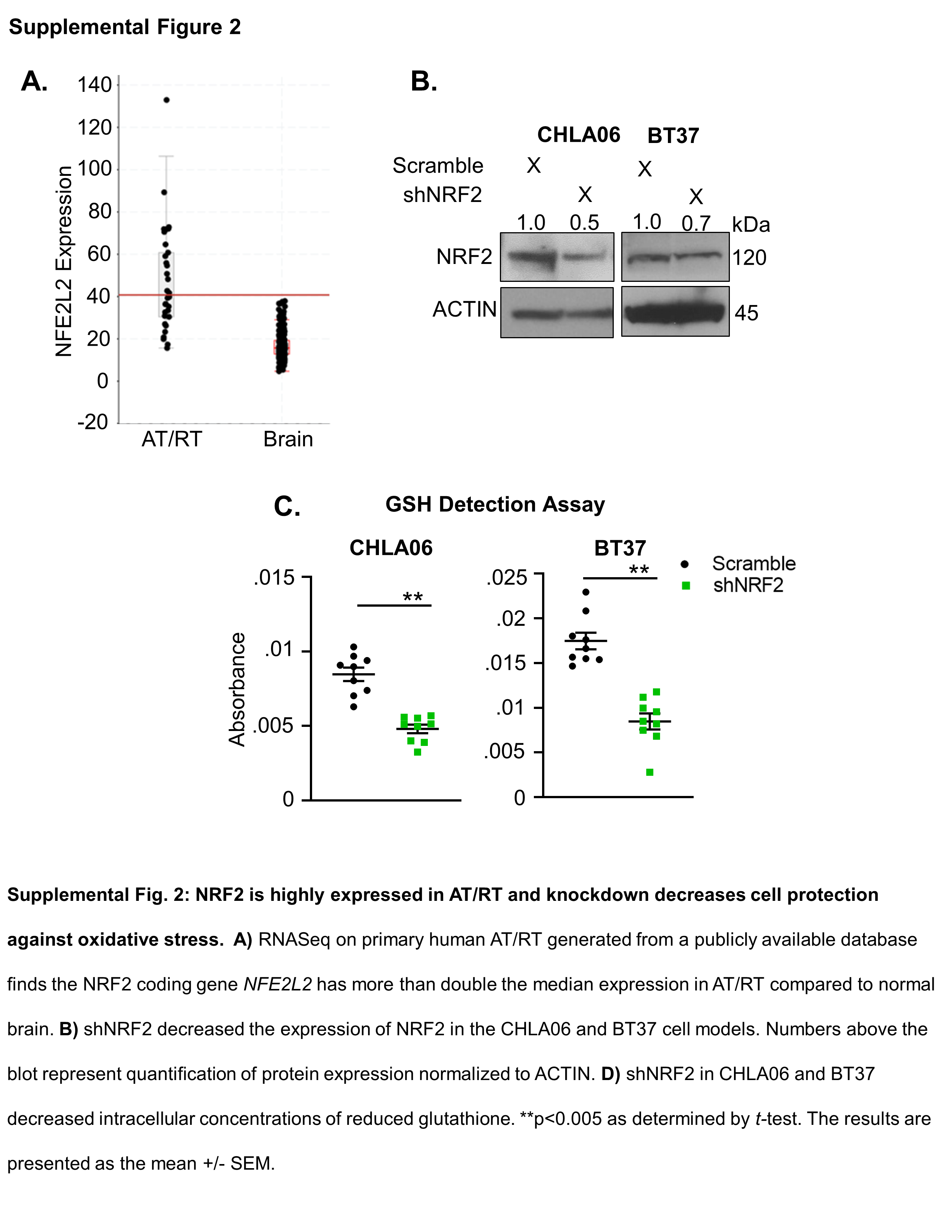

Supplement: Supplementary file 4 — Supplemental Figure 2 [file 41419_2022_4868_MOESM4_ESM.tif]

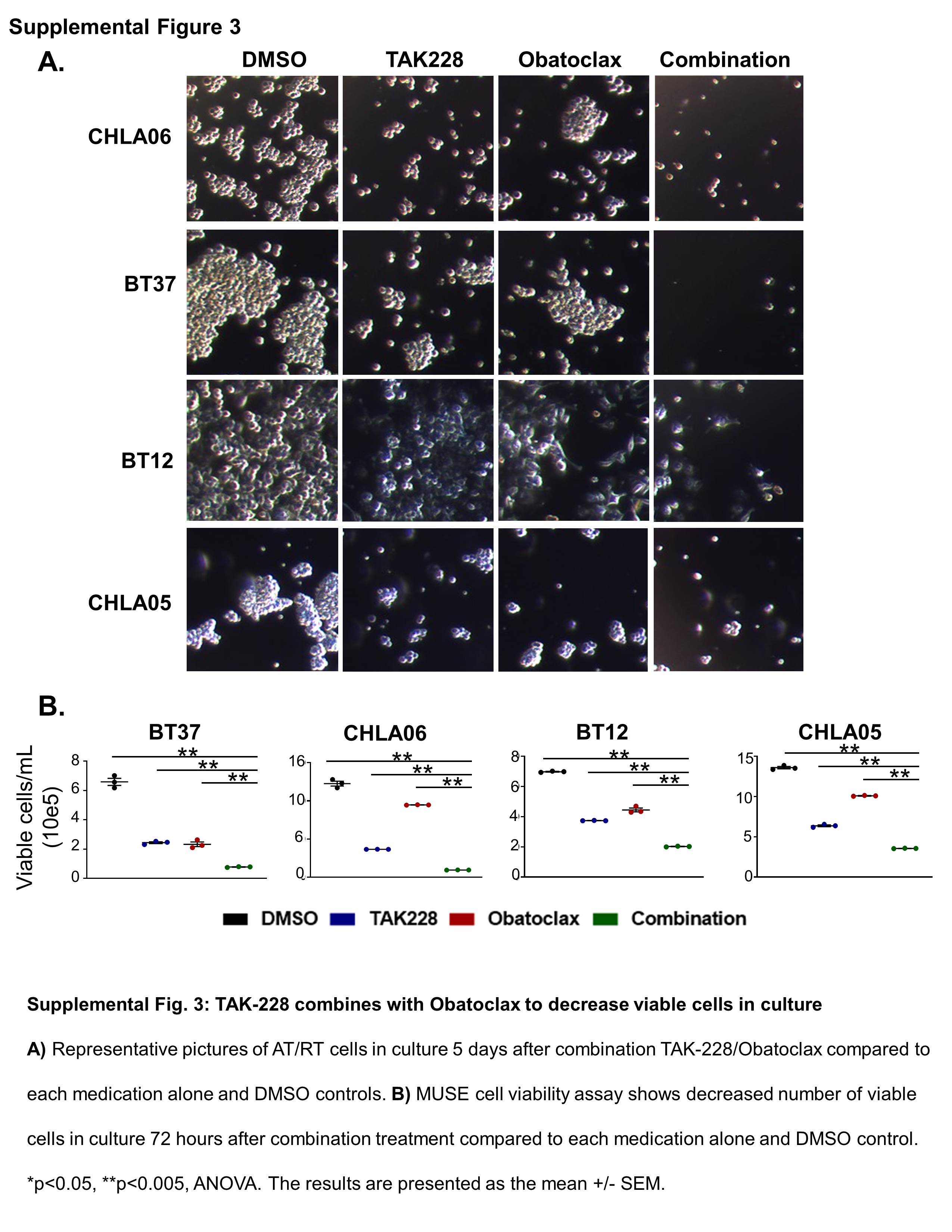

Supplement: Supplementary file 5 — Supplemental Figure 3 [file 41419_2022_4868_MOESM5_ESM.tif]

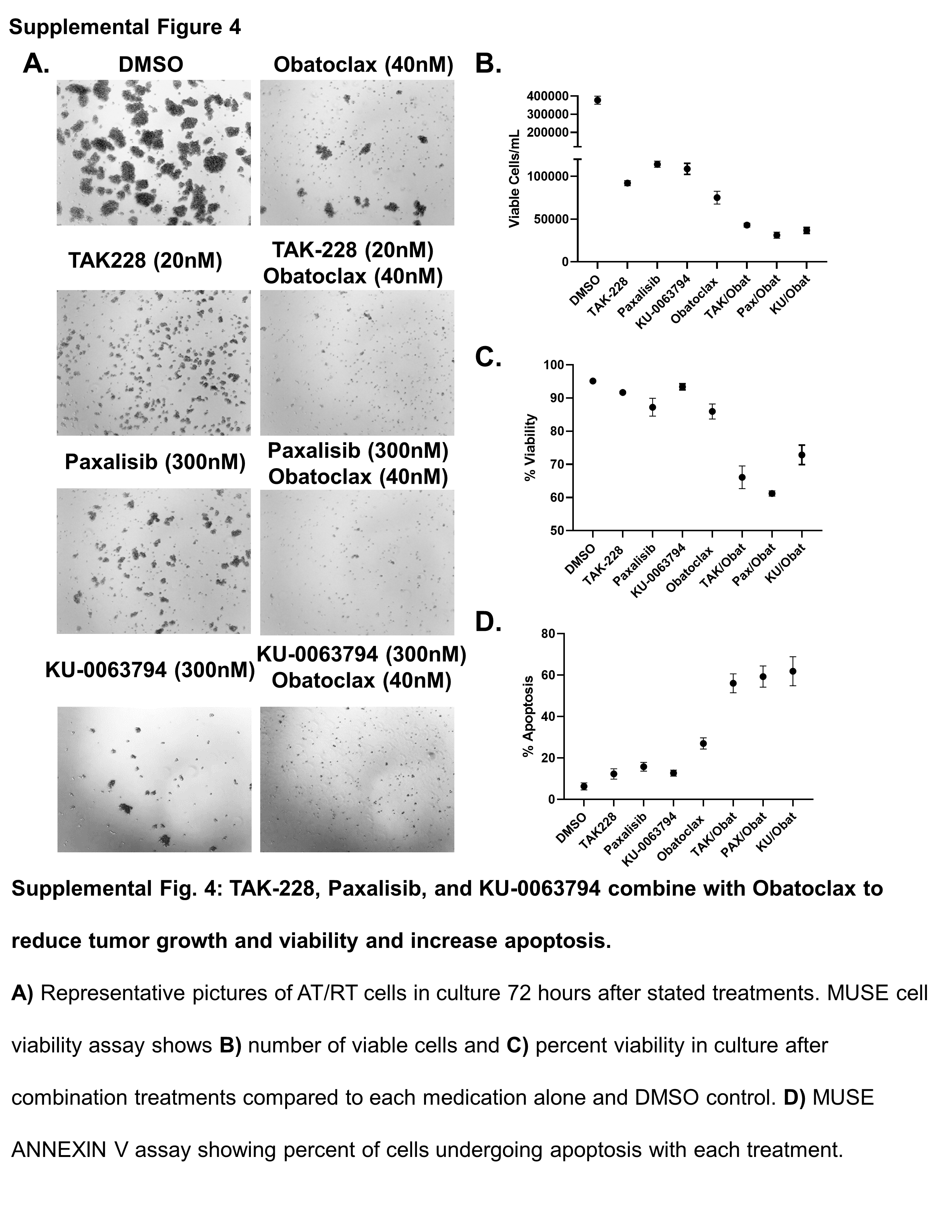

Supplement: Supplementary file 6 — Supplemental Figure 4 [file 41419_2022_4868_MOESM6_ESM.tif]

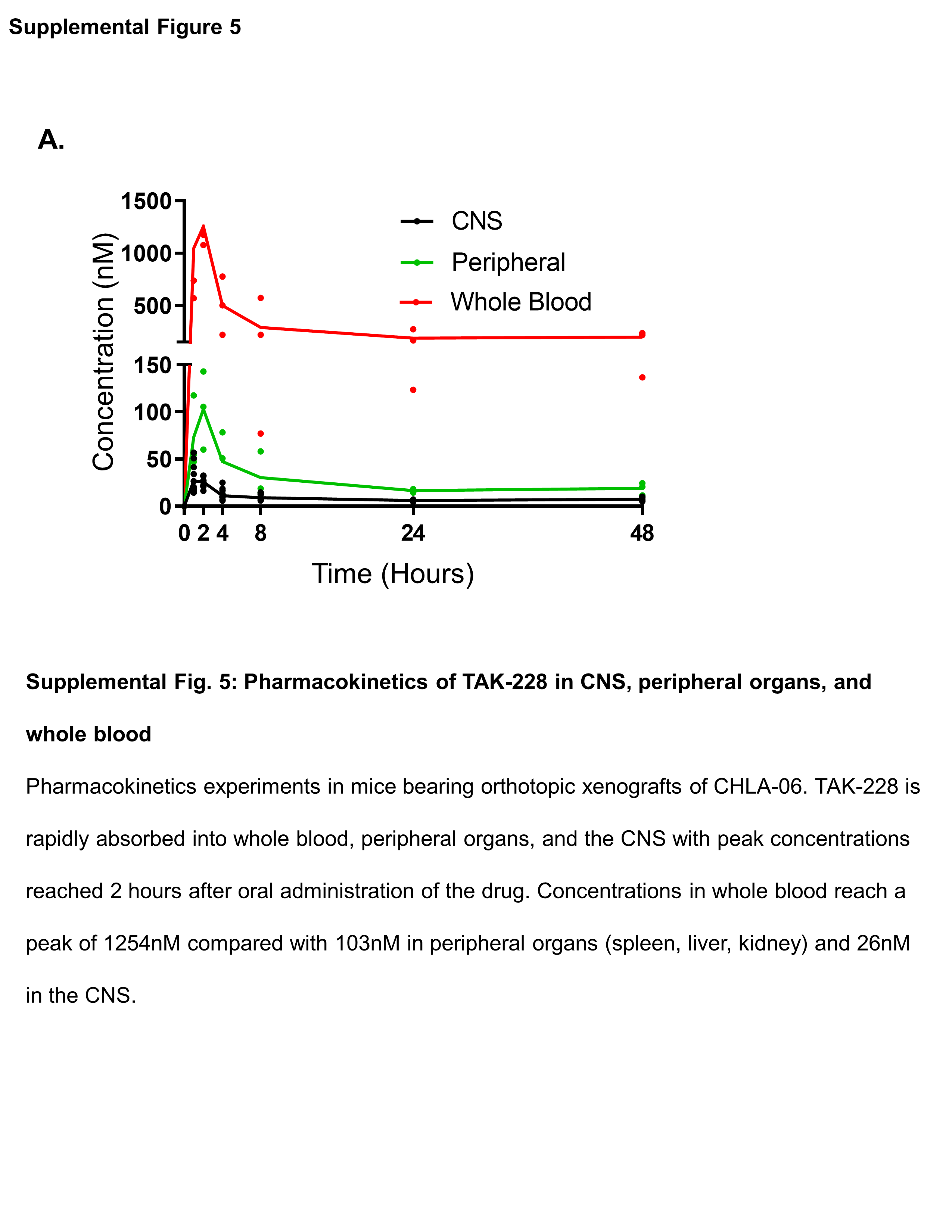

Supplement: Supplementary file 7 — Supplemental Figure 5 [file 41419_2022_4868_MOESM7_ESM.tif]

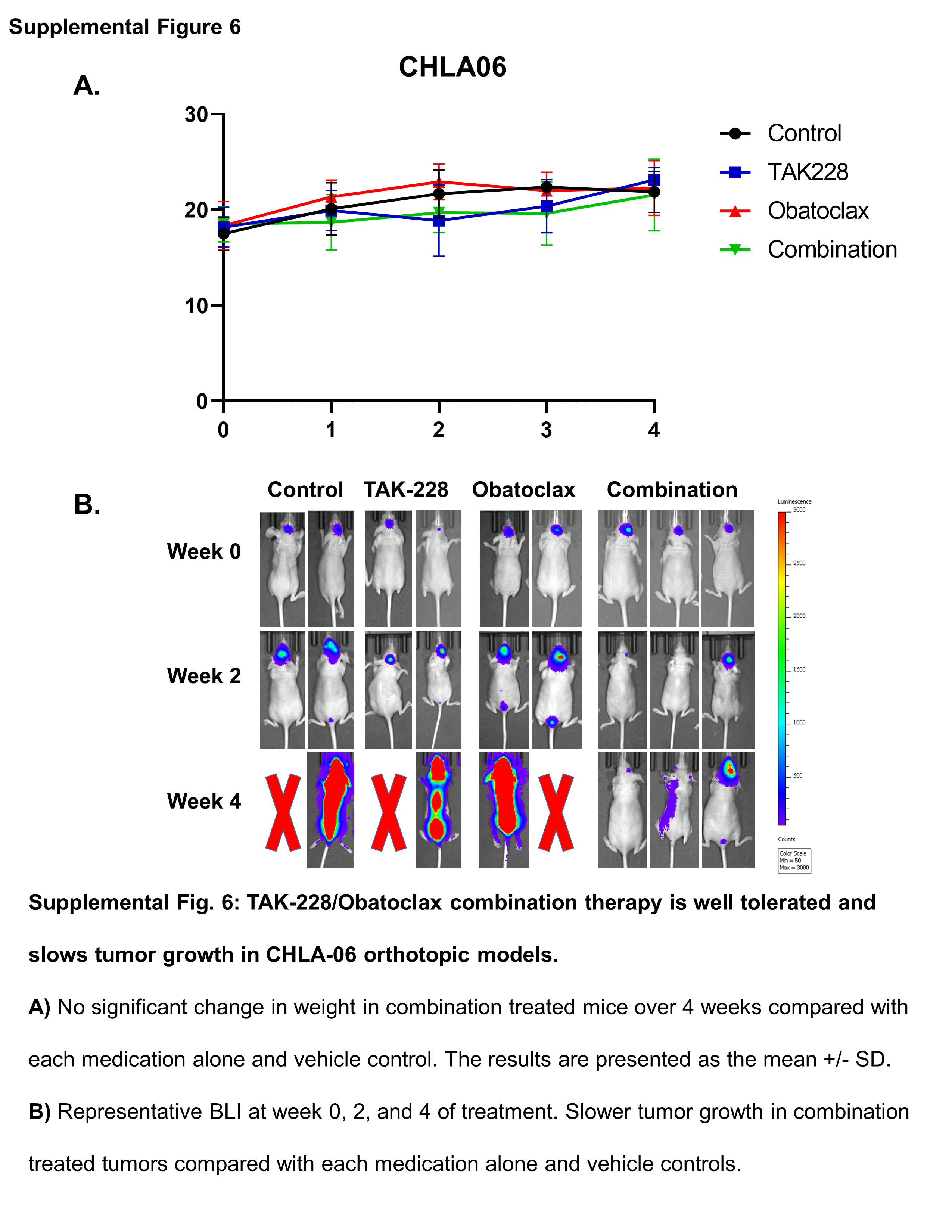

Supplement: Supplementary file 8 — Supplemental Figure 6 [file 41419_2022_4868_MOESM8_ESM.tif]
