# Supplementary material for: Dual mTORC1/2 inhibition compromises cell defenses against exogenous stress potentiating Obatoclax-induced cytotoxicity in atypical teratoid/rhabdoid tumors
Source: Cell Death Dis. 2022 Apr 28;13(4):410. doi: 10.1038/s41419-022-04868-9 (PMC9050713; doi:10.1038/s41419-022-04868-9)
Supplement: Supplementary file 12 — Full WB [file 41419_2022_4868_MOESM12_ESM.pdf]

Fig 1E

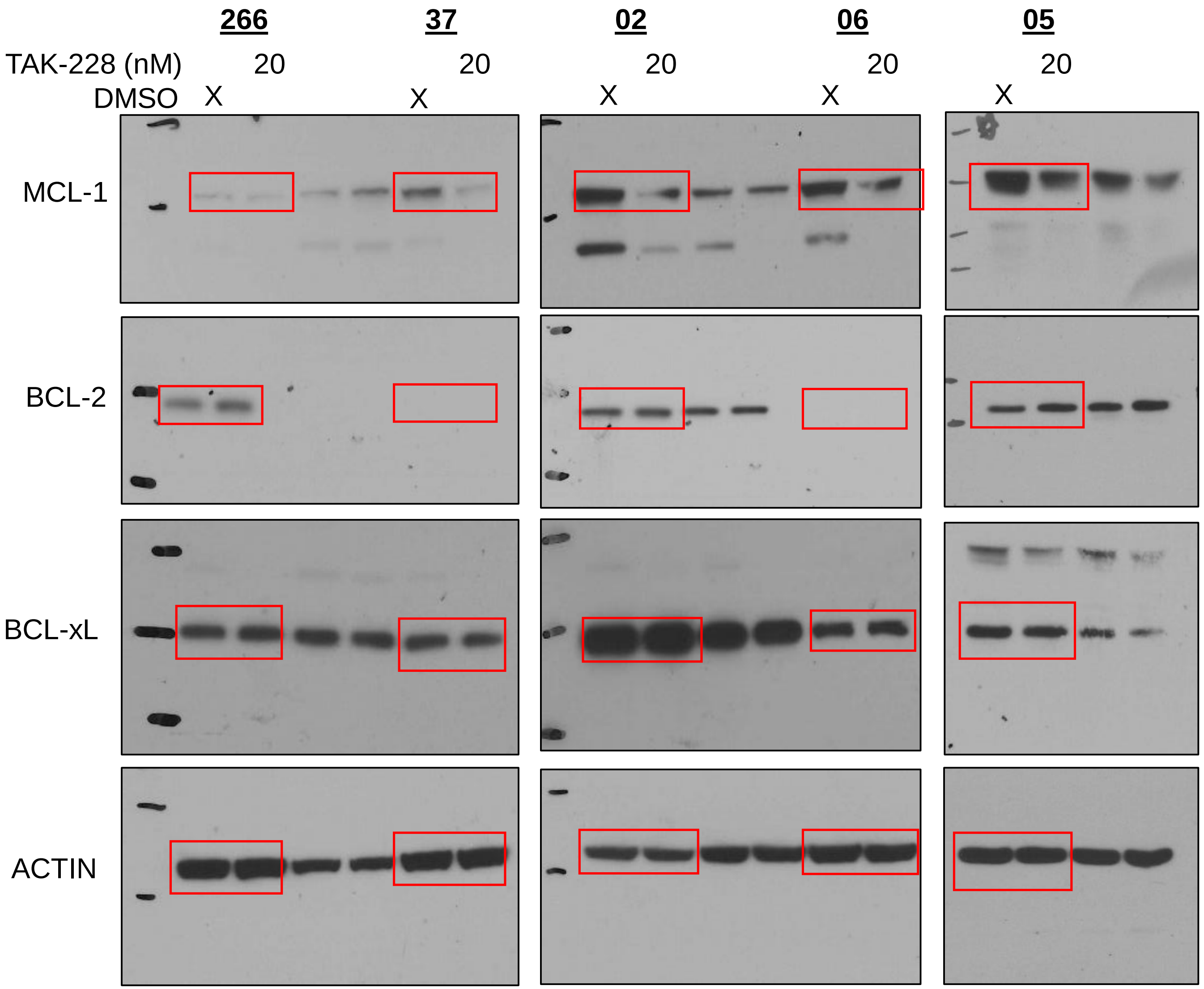

266
37
02
06
05
TAK-228 (nM)
20
20
20
20
20
DMSO
X
X
X
X
X
MCL-1
BCL-2
BCL-xL
ACTIN

Fig 2C

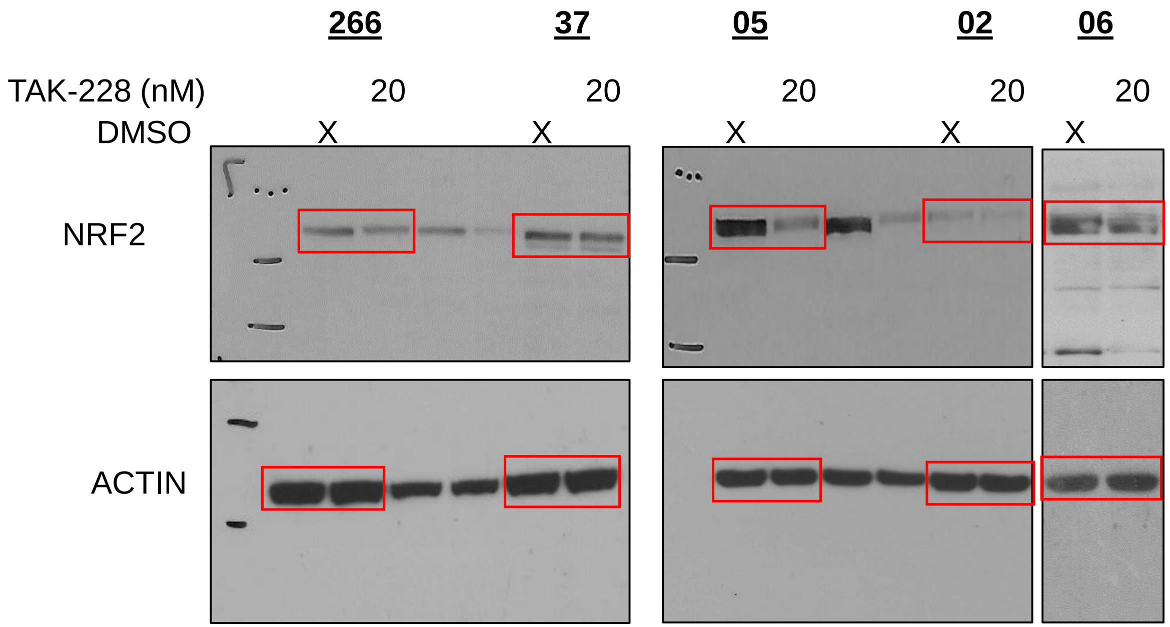

Fig 3D

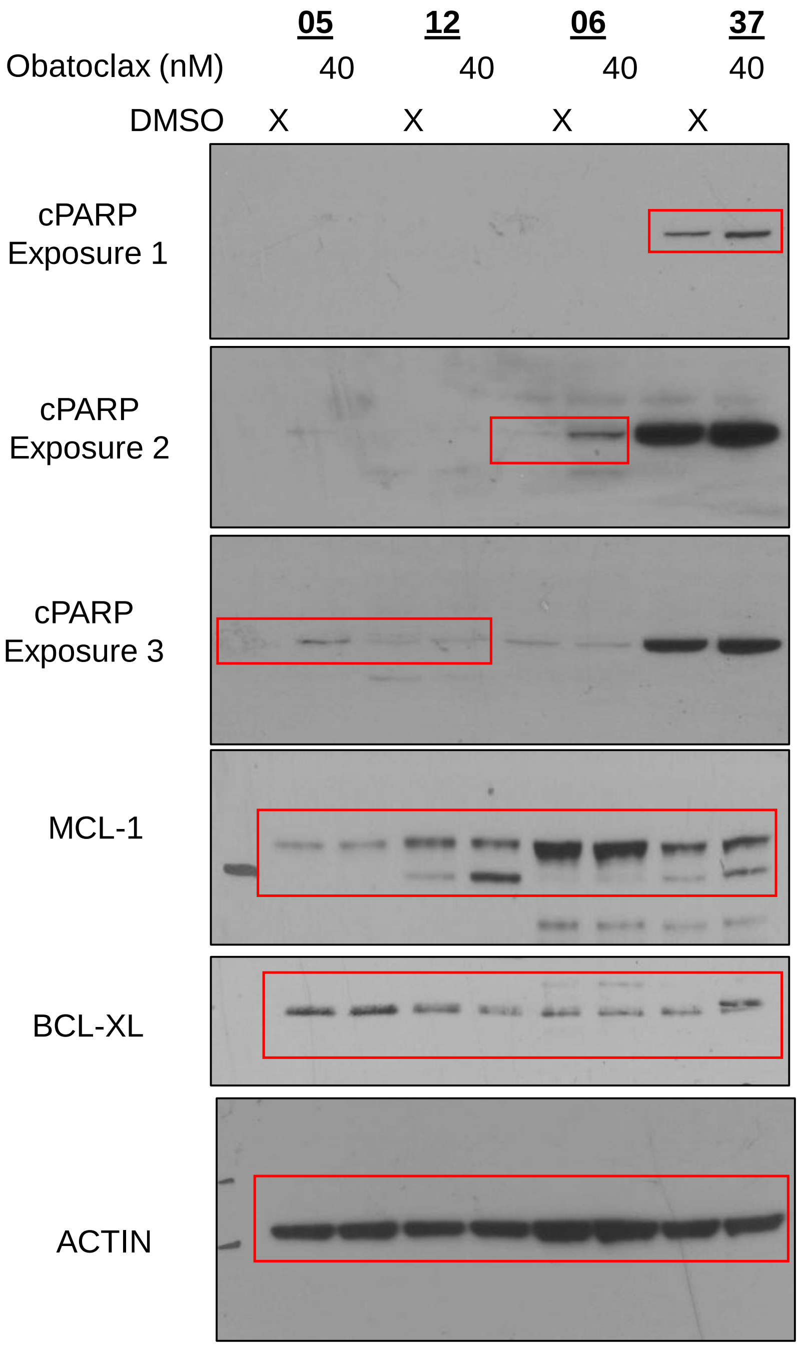

05
12
06
37
Obatoclax (nM)
40
40
40
40
DMSO
X
X
X
X
cPARP
Exposure 1
cPARP
Exposure 2
cPARP
Exposure 3
MCL-1
BCL-XL
ACTIN

Fig 4C

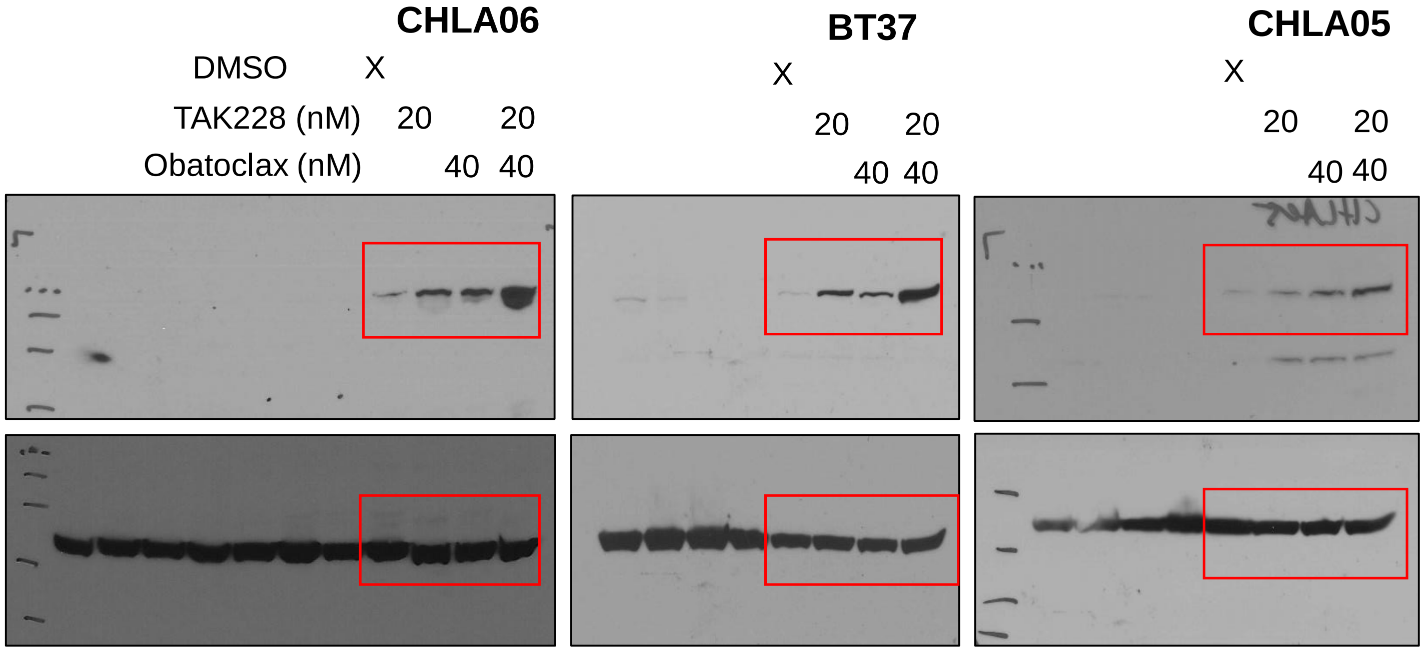
CHLA06
BT37
CHLA05
DMSO X X X
TAK228 (nM) 20 20 20 20 20 20
Obatoclax (nM) 40 40 40 40 40 40

Fig 5D

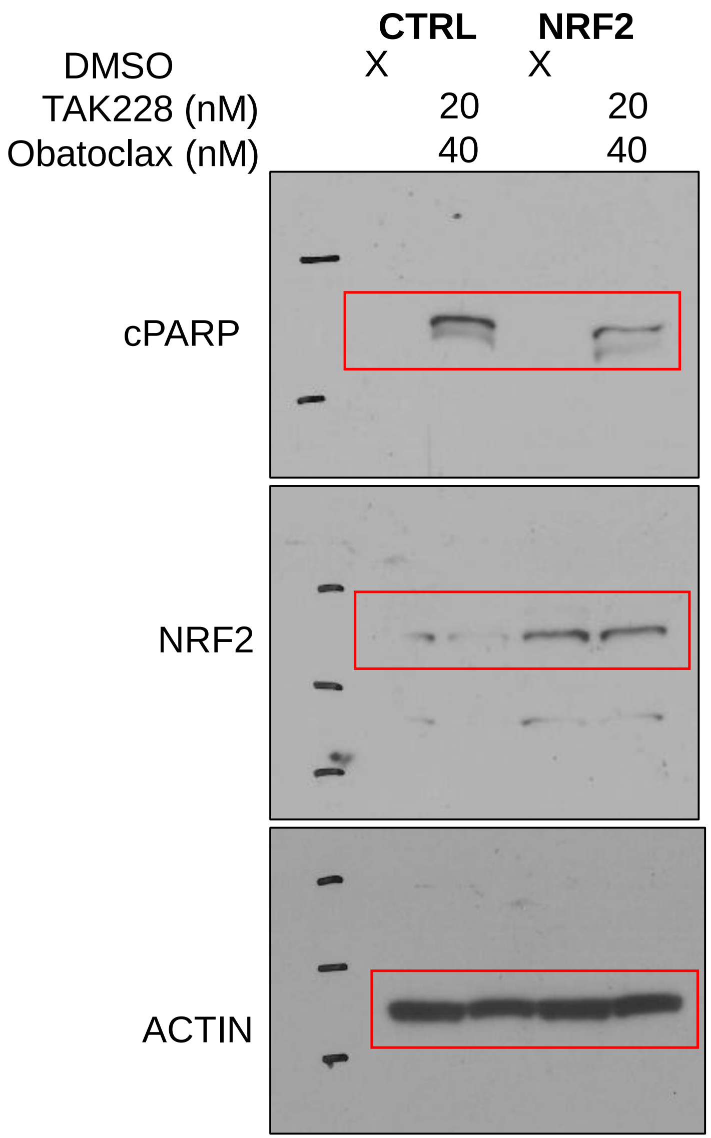

CTRL
NRF2
DMSO
X
X
TAK228 (nM)
20
20
Obatoclax (nM)
40
40
cPARP
NRF2
ACTIN

Fig 6

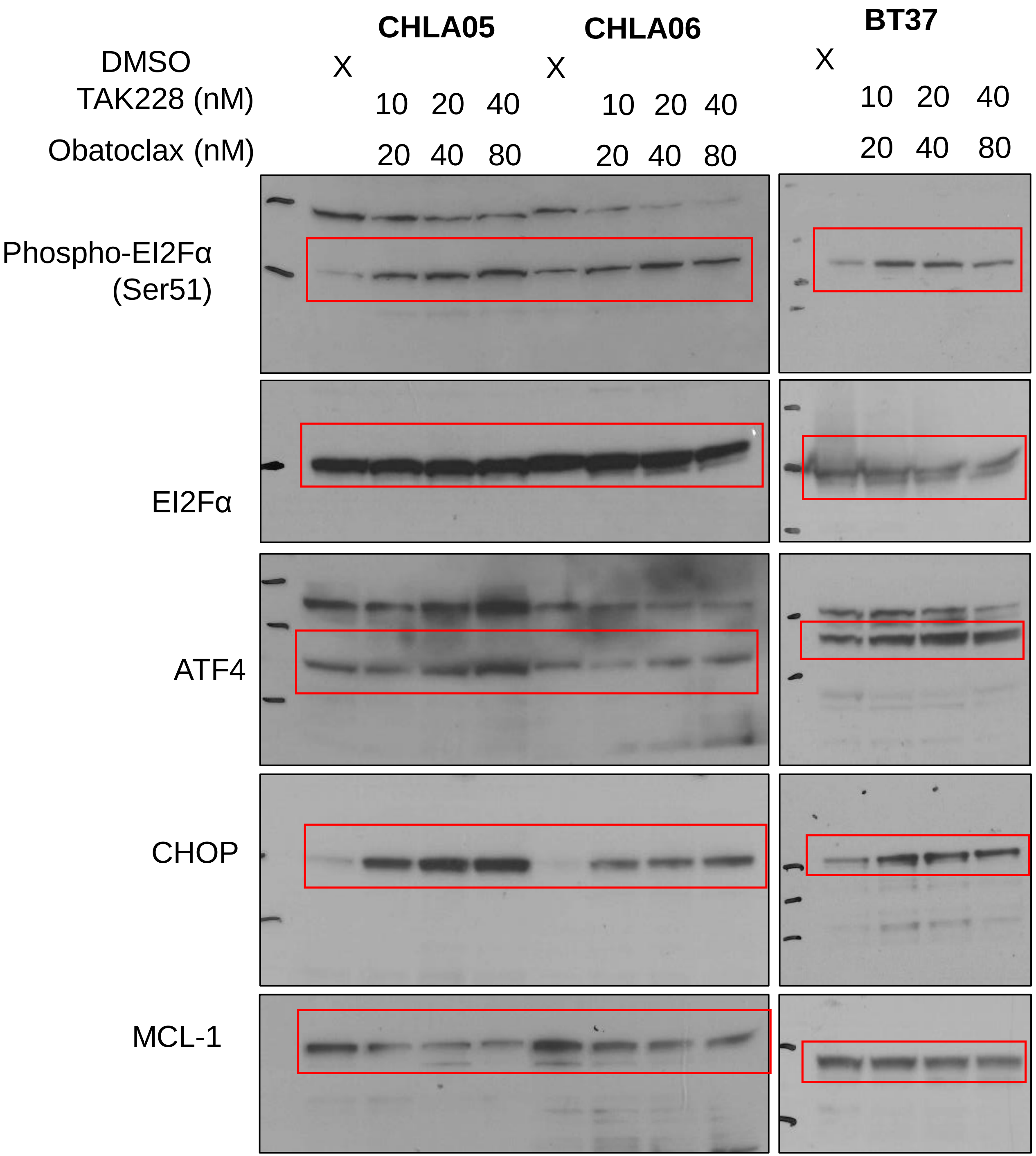
CHLA05
CHLA06
BT37
DMSO
TAK228 (nM)
Obatoclax (nM)
X
10 20 40
20 40 80
X
10 20 40
20 40 80
X
10 20 40
20 40 80
Phospho-EI2Fα (Ser51)
EI2Fα
ATF4
CHOP
MCL-1

Fig 6 (continued)

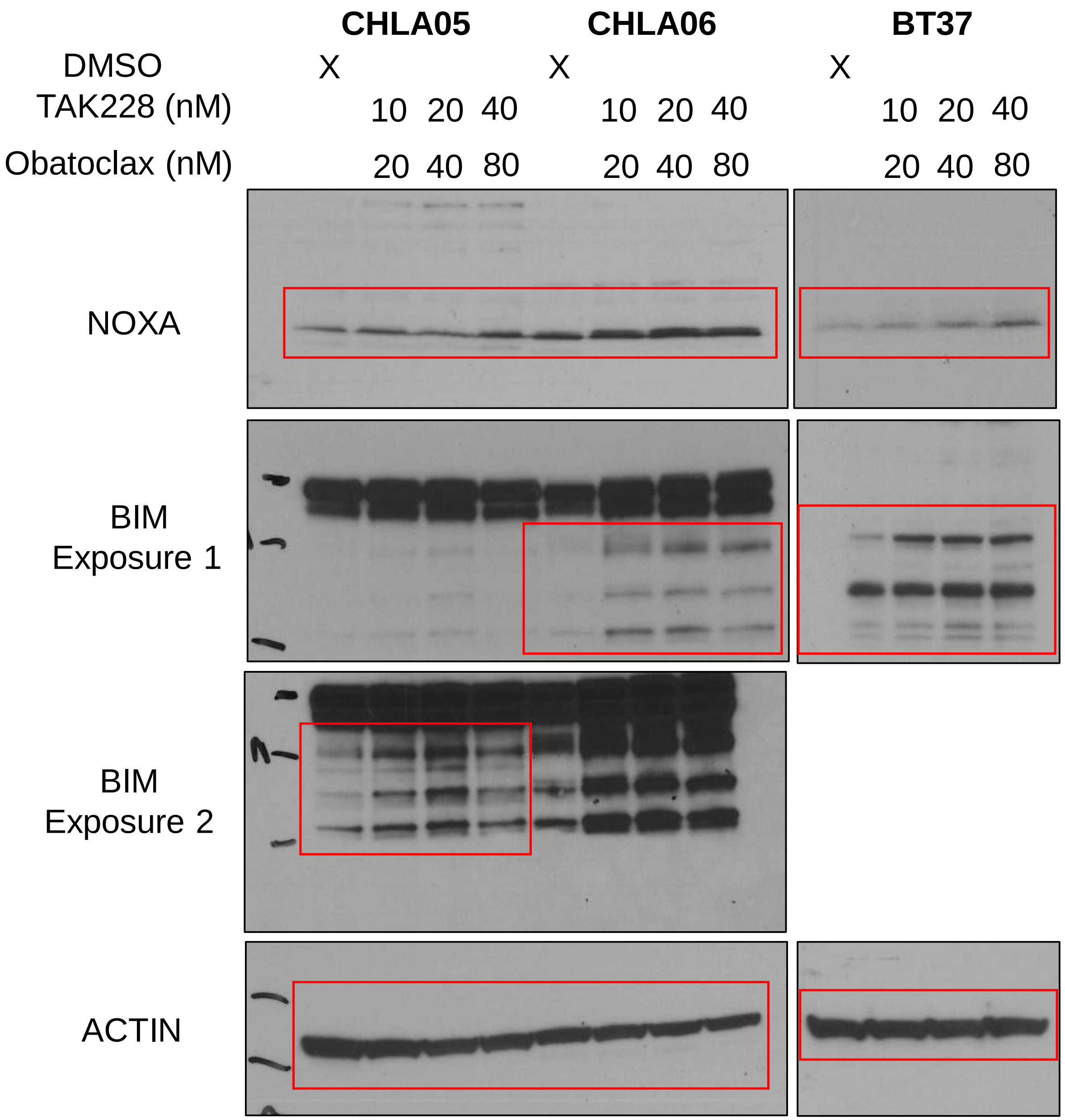

Fig 7C

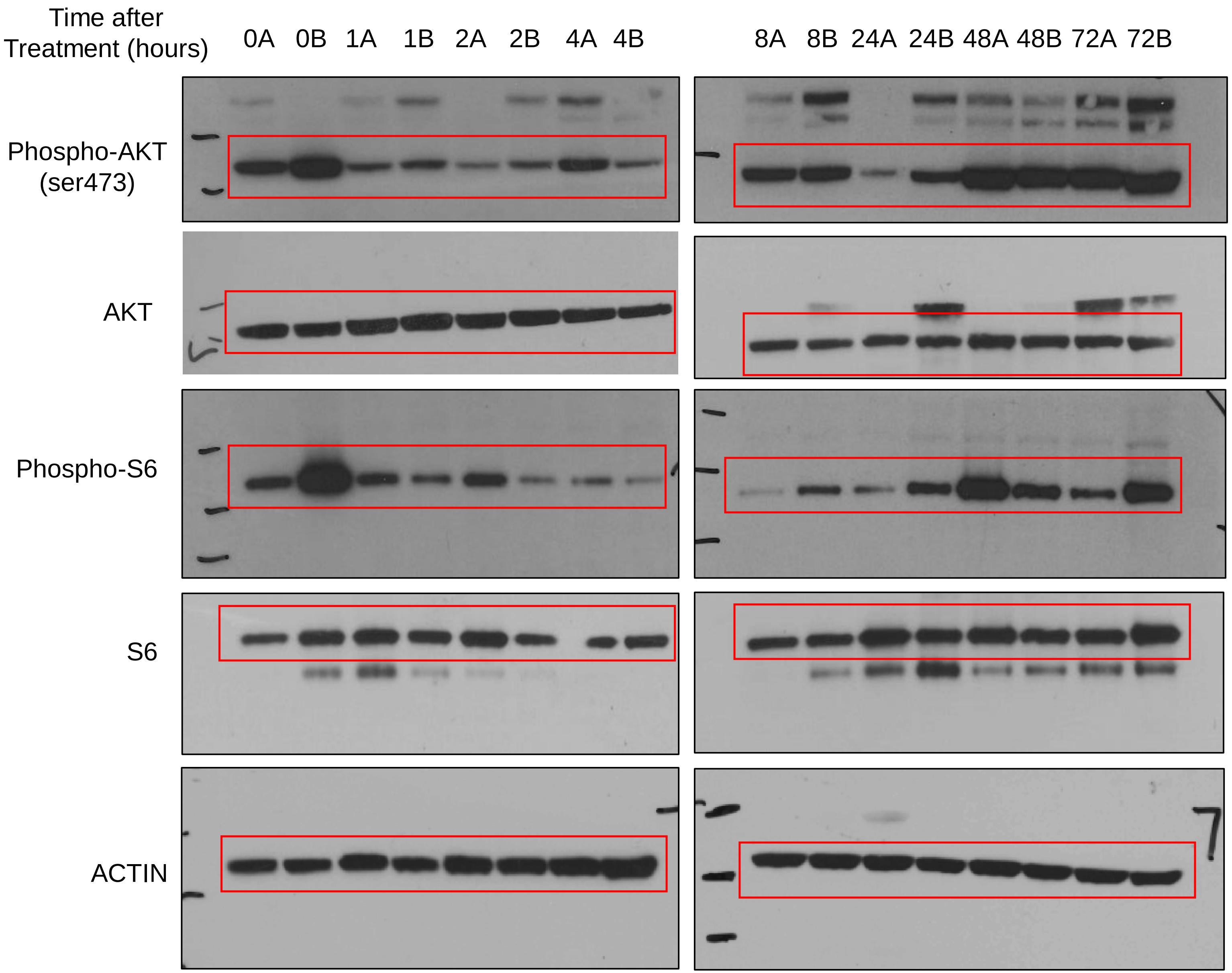

Time after Treatment (hours)
0A 0B 1A 1B 2A 2B 4A 4B
8A 8B 24A 24B 48A 48B 72A 72B
Phospho-AKT (ser473)
AKT
Phospho-S6
S6
ACTIN

Fig 8B

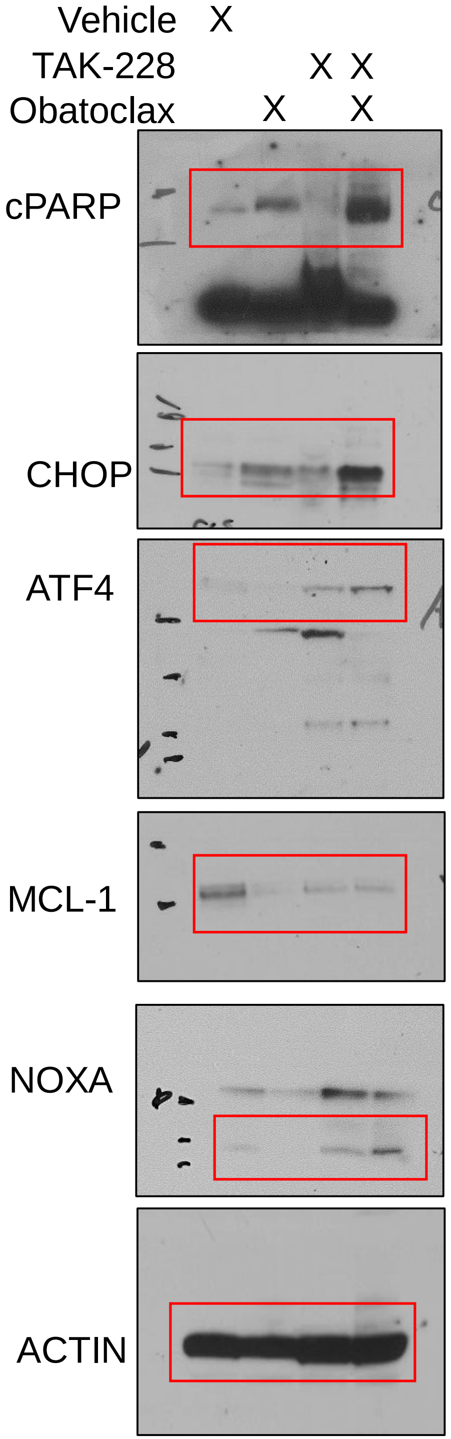
Vehicle X
TAK-228 X X
Obatoclax X X
cPARP
CHOP
ATF4
MCL-1
NOXA
ACTIN
